# BCFL-DAI-PUB: Blockchain-integrated federated learning with dynamic adaptive incentives for privacy-utility balanced credit risk modelling

**DOI:** 10.1371/journal.pone.0361223

**Published:** 2026-10-29

**Authors:** Padmaja Ramachandra, Santhi Vaithiyanathan

**Affiliations:** 1 Vellore Institute of Technology, School of Computer Science and Engineering, Vellore, Tamil Nadu, India; 2 Vellore Institute of Technology, School of Computer Science and Engineering, Chennai, Tamil Nadu, India; King Abdulaziz University, SAUDI ARABIA

## Abstract

Accurate prediction of loan defaulters through collaborative analysis of financial data across multiple institutions is critical for maintaining financial stability and strengthening credit risk management. Although Federated Learning (FL) enables organizations to train models without exchanging raw data, its reliance on a centralized server introduces serious trust concerns, including vulnerability to gradient inversion and membership inference attacks. Blockchain-based Federated Learning (BCFL) addresses these challenges by cryptographically protecting and aggregating model updates through a smart contract, thereby providing a tamper-resistant learning process. In spite of these security enhancements, current BCFL frameworks do not ensure sustained client participation in real-world settings. Moreover, while traditional incentive mechanisms promote truthful participation and discourage free-riding and malicious behavior, they mainly reward model utility and neglect privacy contribution. To mitigate this limitation, we propose BCFL-DAI-PUB, a blockchain-federated learning framework integrating an innovative Dynamic Adaptive Incentive (DAI) mechanism to achieve a Privacy–Utility Balance (PUB). The proposed DAI dynamically applies three complementary incentive components, such as SCAFFOLD-based correction, adaptive differential privacy (DP-Boost), and monetary rewards, to jointly optimize learning utility and privacy protection. This work promotes honest participation, discourages dishonest behavior, and enables rapid recovery from malicious attacks, thereby improving global convergence and overall model effectiveness. The proposed BCFL-DAI-PUB achieves a final AUC of 0.91, improving upon the HE-based BCFL-PS-HER baseline (0.86) by 1.06×. By replacing homomorphic encryption with adaptive differential privacy and combining it with a dynamic incentive mechanism, the proposed framework improves both efficiency and resilience over the baseline, which relies on static reputation-based coordination. Compared with the baseline BCFL-PS-HER model, BCFL-DAI-PUB recovers from malicious disruption 1.82× faster and achieves approximately 1.22× higher MIA resistance, while the baseline incurs approximately 1.09× higher communication overhead and 1.14× higher storage overhead. These results show that BCFL-DAI-PUB improves predictive performance, strengthens privacy protection, reduces system cost, and provides more robust operation in blockchain-enabled federated credit-risk modelling.

## Introduction

A loan defaulter refers to a person who does not pay back the loan amount to the bank. Predicting such defaulters plays an important role in the finance domain to reduce credit risk and maintain financial stability [1]. With the rapid growth of Artificial Intelligence (AI), financial institutions employ AI-driven data models to improve credit risk evaluation [2–4]. However, the success of these models depends largely on access to large, diverse datasets that accurately reflect different types of borrower data. In real-world financial applications, such large and diverse datasets can be obtained through collaboration among financial institutions, but sharing sensitive financial data is often restricted by regulations such as the General Data Protection Regulation (GDPR) [5].

Federated Learning (FL) is an effective approach to address the underlying privacy and compliance issues. FL allows financial institutions to jointly train a shared model without sharing raw data, transmitting only model updates to a central aggregator while preserving privacy and exploiting data heterogeneity [6–8]. However, FL remains vulnerable to inference attacks, such as gradient and membership inference attacks, which allow attackers to reconstruct sensitive data and identify members, respectively [9,10]. Such attacks significantly limit the applicability of FL in sectors like finance and health care, where privacy is of paramount importance.

The integration of Federated Learning (FL) and Blockchain (BC) has resulted in the Blockchain-Federated Learning (BCFL) framework, designed to improve privacy, security, and accountability in collaborative financial settings [11]. Blockchain technology in BCFL offers a decentralized and tamper-resistant ledger for model updates, allowing participants to verify operations without dependence on a central authority. Smart contracts are self-executing programs on the blockchain, enforcing predefined rules and executing actions automatically when specified conditions are satisfied. Together, these blockchain and smart-contract features strengthen collaborative credit risk modeling [12]. Yet, BCFL faces difficulties in maintaining long-term participation and ensuring fairness among institutions with diverse data characteristics and computational capacities.

Further, in decentralized learning settings, participants may exhibit adversarial behaviors, such as free riding or intentionally manipulating the learning process. To deal with these problems, different incentive systems have been suggested [13–18]. These systems usually reward participants based on measurable factors like the amount of data they provide [13], how much they improve the model [14–16], the quality of their data [17], their use of resources [18], or their past reputation.

A significant gap exists in the current incentive mechanism because most techniques reward utility while overlooking privacy contributions. Even when BCFL frameworks employ advanced privacy-preserving techniques, their incentive mechanisms give priority to utility enhancement. This imbalance is problematic because privacy and utility are inversely proportional, and improving one would weaken the other. In financial applications, sacrificing either option entails a higher risk. Therefore, an incentive mechanism that jointly rewards privacy preservation and predictive performance is essential to maintain a stable privacy–utility balance.

To address the underlying challenge, this paper introduces Blockchain-Integrated Federated Learning with a Dynamic Adaptive Incentive Mechanism for Privacy–Utility Balanced (BCFL-DAI-PUB) credit risk assessment model. The framework proposes a dynamic incentive mechanism that adjusts client rewards to balance privacy preservation and predictive utility in credit risk modeling. We summarize our contributions as follows:

1. **Fully Decentralized Preprocessing Pipeline for BCFL:** We introduce a BCFL-compatible preprocessing technique where smart contracts manage global data transformations, remove dependence on trusted coordinators, and provide auditable and consistent execution across clients.
2. **Smart Contract-Enforced Global Feature Selection via Explainable AI:** We propose a novel, blockchain-governed feature selection mechanism that leverages SHAP values to collaboratively identify the most informative global feature subset.
3. **Privacy-Preserving Synthetic Data Generator Using Transfer Learning and Diffusion Model:** Our novel synthetic data generator speeds up convergence in challenging non-IID environments by leveraging a Self-Supervised Learning Tabular Transformer as a way to transmit learning to the diffusion model. Also, it makes privacy protections stronger against inference attacks, such as membership inference attacks and gradient inference attacks, by adding Gaussian noise at the optimizer level.
4. **Dynamic Adaptive Incentive Mechanism (DAI) for Balanced Privacy-Utility Framework:** We develop a DAI mechanism that dynamically adjusts the Global Correction Error, Adaptive Privacy Budget, and monetary incentives to maintain privacy–utility equilibrium while safeguarding the global model against malicious and free-rider behavior.
5. **Experimental Assessment:** Our empirical research examines the efficacy and robustness of the proposed framework by analyzing five distinct simulated client behaviors using the Dynamic Adaptive Incentive Mechanism. In addition, the global model’s performance, recovery speed from malicious attacks, and convergence behavior are evaluated across training rounds under both fully honest and dishonest settings. Lastly, the privacy robustness is assessed against Membership Inference Attacks (MIA) and Gradient Inference Attacks (GIA).

## Related work

Table 1 compares representative studies in federated learning (FL), blockchain-based federated learning (BCFL), and BCFL with incentive mechanisms, with emphasis on their relevance to credit risk assessment. The comparison shows that recent research has moved beyond basic privacy-preserving collaboration toward decentralized trust management, explainability, privacy-leakage resilience, and incentive design. These directions, however, remain only partially explored in finance-oriented settings.

**Table 1:** Comparative analysis of federated learning frameworks for credit risk assessment with respect to decentralization, incentive design, and privacy–utility awareness.

S.NO.	Paper	Year	Federated Learning	Block Chain	Incentive Mechanism	Credit Risk Assessment	Utility-Aware Incentives	Privacy-Aware Incentives
Federated Learning for Credit Risk Assessment								
1	[19]	2024	Yes	No	No	Yes	NA	NA
2	[20]	2025	Yes	No	No	Yes	NA	NA
Federated Learning integrated with Blockchain for Credit Risk Assessment								
3	[21]	2024	Yes	Yes	No	Yes	NA	NA
4	[22]	2024	Yes	Yes	No	Yes	NA	NA
Federated Learning and/or Blockchain, Incentive Mechanisms Evaluated on Non-Credit-Risk Datasets								
5	[23]	2021	No	Yes	Yes	No	NA	NA
6	[24]	2022	Yes	Yes	Yes	No	Yes	No
7	[25]	2024	Yes	Yes	Yes	No	No	I&NEP
8	[26]	2025	Yes	Yes	Yes	No	Yes	I&NEP
9	[27]	2025	Yes	Yes	Yes	No	Yes	I&NEP
10	[28]	2025	Yes	Yes	Yes	No	Yes	NA
11	[29]	2025	Yes	No	Yes	No	Yes	I&NEP
12	[30]	2025	Yes	No	Yes	No	Yes	No
Federated Learning integrated with Blockchain and Incentive Mechanism on Credit Risk Assessment								
13	[31]	2024	Yes	Yes	Yes	Yes	Yes	I&NEP
14	Proposed	2025	Yes	Yes	Yes	Yes	Yes	Yes

NA: Not Applicable I&NEP: Implemented but Not Empirically Proved.

Recent studies have shown that federated learning can support credit risk assessment without direct data centralization. Li et al. [19] address non-IID financial default data and communication overhead by combining a dynamic receptive field mechanism with a multi-fusion strategy for aggregating local and global features, reducing communication rounds by approximately 80%. Their framework does not, however, add privacy mechanisms beyond keeping raw data local. Ramachandra and Vaithiyanathan [20] strengthen privacy and predictive performance for loan default prediction through autoencoders, a federated Wasserstein GAN with a differentially private optimizer, and CTGAN-based oversampling, reporting a privacy–utility trade-off with 95% accuracy against 82% for a non-federated setup. Their framework still relies on a central server for coordination, and it includes neither verifiable aggregation nor an incentive mechanism.

Conventional federated learning also remains exposed to gradient-leakage attacks, through which adversaries may reconstruct or infer sensitive client information from shared model updates, and centralized coordination introduces a single point of failure. These limitations have encouraged the integration of blockchain with federated learning. By replacing centralized coordination with a blockchain, BCFL enables tamper-resistant model exchange, verifiable aggregation, and auditable training records, which are particularly valuable in regulated financial applications where transparency, accountability, and trust are essential.

Within the financial domain, recent BCFL studies have begun to address these concerns. Baabdullah et al. [21] apply BCFL to credit card fraud detection, a credit-related financial risk task, across multiple banks and show that it can improve detection performance while preserving data privacy. They also identify unresolved challenges, including real-time detection, scalability, false-alarm rates, and resilience against evolving attacks. Jovanovic et al. [22] propose a conceptual framework that integrates Explainable AI into a BCFL-based credit-scoring process, in which dedicated miners generate SHAP-based explanations and model and explanation parameters are stored off-chain with their hashes recorded on the blockchain. This strengthens transparency and auditability, although the framework is not empirically evaluated and includes no incentive mechanism.

Although BCFL improves trust, security, and transparency, it does not by itself prevent free-riding or malicious participation. Low-quality or adversarial local updates can still reduce the reliability, convergence, and final performance of the global model. Recent research has therefore focused on incentive mechanisms that reward meaningful participation and discourage dishonest behavior.

Most existing incentive mechanisms for blockchain-based federated learning (BCFL-IM), however, have been evaluated on general-purpose benchmarks, mainly image datasets such as MNIST and CIFAR-10, rather than on financial credit risk data. This leaves a gap between advances in decentralized incentive design and their application to credit risk assessment.

Early studies established the structure of blockchain-supported incentive allocation. Song et al. [23] proposed a Proof-of-Contribution consensus mechanism in which user contribution values determine block-producer selection; the work targets intellectual property protection and does not involve federated learning. Pandey et al. [24] proposed FedToken, a token-based incentive framework in which clients are rewarded through a Shapley value-derived contribution score under a fixed budget. Li et al. [25] proposed PPBFL, which combines a Proof-of-Training-Work consensus mechanism that rewards nodes for computational effort in local training with ring signatures and differential privacy to protect shared updates.

More recent studies emphasize fairness, trustworthiness, and strategic incentive allocation. Chen et al. [26] proposed FairReward, which draws on Adams’ equity theory to distribute rewards by jointly considering clients’ self-reported expected rewards, their contributions, and their reputations. Chen et al. [27] proposed πFL, which values client data through Shapley-based scoring under secure multi-party computation and uses a multi-signature smart contract to guarantee atomic reward payment, so that the server cannot refuse payment.

Incentive design has also advanced through formal economic and game-theoretic models. Tang et al. [28] model incentives in blockchain-based federated learning as a two-tier Stackelberg game, in which the model owner leads the training terminals and multiple miners lead the terminals in a multi-leader multi-follower game. Beyond blockchain-based settings, Yao et al. [29] develop a contract-theoretic incentive mechanism for semi-asynchronous federated learning that accounts for clients’ multidimensional private information, namely data quality, computational resources, and privacy preferences. Zhang et al. [30] address foundation model-enabled cross-silo federated learning, combining dynamic participant screening, a cost–benefit contribution metric, and a two-stage Stackelberg game to suppress knowledge hoarding and free-riding. Taken together, these studies show a growing preference for adaptive, utility-aware incentive design.

Finance-oriented BCFL-IM research remains scarce. The closest work is that of Yang et al. [31], who combine blockchain, homomorphic encryption, and a reputation mechanism, with a reward layer for well-performing edge nodes, in a general Industrial IoT federated learning framework. Their simulations include a loan dataset alongside a house-price dataset, but the framework is not designed for credit risk assessment. Their rewards follow reputation-based contribution evaluation and are not explicitly conditioned on a privacy–utility trade-off, even though credit risk assessment demands both strong predictive performance and protection against privacy leakage.

Overall, the literature shows clear progress in federated credit risk modelling, blockchain-based decentralization, and adaptive incentive design. To the best of our knowledge, however, no existing study jointly addresses predictive utility, privacy preservation, and adversarially robust incentive allocation within a single blockchain-based federated framework for credit risk assessment. This gap motivates the proposed BCFL-DAI-PUB framework, which introduces a Dynamic Adaptive Incentive mechanism that explicitly balances privacy and utility, encourages sustained honest participation, and supports rapid detection and recovery from malicious and free-riding behavior for robust decentralized credit risk modelling.

## Materials and methods

This section presents the materials and methods required to implement and assess the proposed framework.

### Materials

#### Data source and description.

The proposed study uses the Home Credit Default Risk dataset from Kaggle [32], a widely used benchmark for credit-risk assessment and lending decision analysis. The dataset contains 307,511 client records and 122 features, including 106 numerical attributes and 16 categorical attributes. The binary target variable, TARGET, represents loan status, where 1 denotes default and 0 denotes successful repayment.

Table 2 clearly demonstrates that in the original dataset, 24,825 records belong to target = 1 and 282,686 records belong to target = 0, indicating substantial class imbalance in favour of the non-default class. To address this imbalance, we applied oversampling to the minority class before model training. This step increased the dataset size to 565,372 records and produced a balanced class distribution, with 282,686 records in each class. We used this balanced dataset for all subsequent experiments.

**Table 2:** Dataset description.

Description	Value
Original number of records	307,511
Original records with target = 1	24,825
Original records with target = 0	282,686
Number of records after oversampling	565,372
Records with target = 1 after oversampling	282,686
Records with target = 0 after oversampling	282,686
Number of categorical attributes	16
Number of numerical attributes	106
Number of target attributes	1
Target attribute	Loan Defaulter (1) / Not a Loan Defaulter (0)

### Methods

Following the presentation of the necessary materials, the methods subsection details the specific methods and techniques employed in the development and implementation of our framework.

#### Federated learning.

Federated Learning (FL) is a distributed learning paradigm that enables a set of *N* clients, $C=\{C_{1},C_{2},...,C_{N}\}$, to collaboratively train a global model $w\in {\mathbb{R}}^{d}$ without sharing their private datasets.

Each client $C_{i}$ locally stores a dataset as in Equation 1:


$$\begin{array}{c} D_{i}=\{(x_{j},y_{j}){\}}_{j=1}^{n_{i}}, \end{array}$$ (1)

while the global optimization objective is formulated as in Equation 2:


$$\begin{array}{c} \min_{w\in {\mathbb{R}}^{d}}F(w), \end{array}$$ (2)

where $F(w)=\sum_{i=1}^{N}\frac{n_{i}}{n}F_{i}(w)$, $n=\sum_{i=1}^{N}n_{i}$, and each client’s local loss is defined by Equation 3:


$$\begin{array}{c} F_{i}(w)=\frac{1}{n_{i}}\sum_{(x_{j},y_{j})\in D_{i}}\ell (w;x_{j},y_{j}). \end{array}$$ (3)

During FL training, Each client trains the model with their own data and transmits the updates $\Delta w_{i}$ to the aggregation, which computes the global weighted average to derive the global model. Thus, FL improves learning efficiency while preserving privacy.

#### Blockchain-integrated federated learning (BCFL).

Blockchain-Integrated Federated Learning (BCFL) enhances conventional Federated Learning by replacing the vulnerable central aggregator with a blockchain and smart contracts. BCFL allows the system to operate in a fully decentralized manner while securely recording and validating model updates in a transparent way. The reliability of BCFL is guaranteed through a distributed ledger, consensus mechanisms, and smart contracts [33,34].

#### Incentive mechanism in BCFL (BCFL-IM).

Although BCFL assures decentralized trust, it does not inherently guarantee honest or high-quality participation. Because local training incurs computational costs, rational clients may attempt to free-ride or submit low-quality updates, which degrade overall global model performance. Therefore, incentive mechanisms are necessary for promoting sustained participation, ensuring contribution quality, discouraging free-riding, and mitigating adversarial behavior [35]. Existing schemes include contract-theoretic [36,37], reputation and auction-based [38,39], and game-theoretic approaches [40]. Nevertheless, these approaches are generally static and specific to particular domains. To address these limitations, this work introduces a Dynamic Adaptive Incentive (DAI) mechanism that adjusts client rewards in response to real-time performance and privacy, suppresses malicious and free-riding behavior, and strengthens convergence reliability in decentralized financial environments.

#### Diffusion models for privacy-preserved data generation.

To add privacy to the BCFL framework, this work adopts a diffusion model as the core generative architecture due to its stability and high-quality synthetic data generation. Unlike Generative Adversarial Networks (GANs), which may suffer from mode collapse and training instability, Variational Autoencoders (VAEs) often produce overly smooth samples and may have limited ability to capture complex data distributions. Diffusion models use an iterative denoising procedure to learn the underlying data structure and produce high-quality synthetic samples, which makes them especially suitable for privacy-preserving data generation. The proposed framework employs the Tabular Denoising Diffusion Probabilistic Model (TabDDPM) [41–43], which is specifically engineered for heterogeneous tabular datasets. TabDDPM works with two diffusion processes: 1) Forward noising transform real data into noise, and 2) Reverse denoising reconstructs high-quality synthetic data while preserving the privacy.

#### Dual-layered privacy defense in diffusion model training.

Diffusion models in BCFL are susceptible to reconstruction attacks because even though the model updates that are recorded on the blockchain offer transparency, their immutable nature raises privacy threats. To mitigate these threats, the proposed work incorporated differential privacy [44,45] at the optimizer level to provide client-side privacy assurance in a fully decentralized and publicly verifiable environment. The Gaussian mechanism is used because it is stable when working with high-dimensional tabular data, as shown in Equation 4:


$$\begin{array}{c} ℳ(D)=f(D)+𝒩(0,\sigma^{2}𝐈), \end{array}$$ (4)

where the noise scale satisfies as shown in Equation 5:


$$\begin{array}{c} \sigma \geq \frac{\Delta_{2}f\cdot \sqrt{2\ln(1.25/\delta )}}{\varepsilon }. \end{array}$$ (5)

Thus, the framework establishes a robust, dual-layer protection by integrating blockchain-based federation with differential privacy at the optimizer level as:

- **Decentralized Structural Protection:** Blockchain-powered federation aggregates updates without centralizing raw data, eliminating single points of failure and neutralizing server-side leakage risks.
- **Mathematical Generative Protection:** Optimizer-level differential privacy prevents sample memorization and blocks gradient inversion and reconstruction attacks.

Together, these layers ensure that the proposed BCFL-DAI-PUB framework achieves strong privacy, robust learning, and resilience against advanced adversarial threats.

## Threat models and evaluation metrics

The proposed Blockchain-Federated Learning (BCFL) framework is evaluated across two complementary threat dimensions. The first focuses on privacy resilience, examined through the execution of Gradient Inversion Attacks (GIA) and Membership Inference Attacks (MIA). The second assesses robustness against adversarial behavior by simulating malicious client activities, including label-flipping attacks. In addition, the overall effectiveness of the framework is measured using standard performance evaluation metrics.

### Gradient inversion attack (GIA)

We evaluate the privacy robustness of the proposed BCFL framework against state-of-the-art Gradient Inversion Attacks (GIAs), which attempt to reconstruct private client training samples using only observed gradients [46]. We adopt the widely accepted honest-but-curious adversary model, in which the attacker has full access to public model parameters but cannot observe individual client gradients; only aggregated updates are available. The attacker executes the GIA pipeline with the following objective function (Equation 6) to minimize the discrepancy between the attacker’s synthetic gradient and the true gradient ($g_{i}$) [47]


$$\begin{array}{c} ℒ(\hat{x})=\underset{\text{analytical gradient matching}}{\underbrace{\|\nabla_{\theta }ℒ(f_{\theta }(\hat{x});y)-g_{i}{\|}_{2}^{2}}}+\lambda_{\text{TV}}\cdot \text{TV}(\hat{x})+\lambda_{\text{range}}\cdot \sum_{j}[\max(0,{\hat{x}}_{j}-u_{j}{)}^{2}+\max(0,l_{j}-{\hat{x}}_{j}{)}^{2}] \end{array}$$ (6)

Here, TV denotes the Total Variation regularizer, while $l_{j}$ and $u_{j}$ enforce valid feature ranges for tabular attributes. The quality of the reconstructed samples is quantified using the following metrics:

- **Mean Absolute Error (MAE):** We measure the average error across features, computed after inverse preprocessing, using the formula presented in Equation 7.

$$\begin{array}{c} \text{MAE}=\frac{1}{d}\sum_{j=1}^{d}|{\hat{x}}_{j}-x_{j}| \end{array}$$ (7)

- **Attribute Inference Accuracy (AIA):** We measure the success of inferring sensitive categorical attributes using the Equation 8:

$$\begin{array}{c} AIA=1\{\hat{a}=a^{*}\}. \end{array}$$ (8)

- **Downstream Task AUC:** We **train** an XGBoost classifier using only the reconstructed samples $\left\{{\hat{x}}_{i},y_{i}\right\}$ as training data and **evaluate** AUC on the official held-out test set. Higher AUC **indicates** that reconstructed records preserve the predictive utility of the original private labels.

Lower MAE and higher AIA/AUC indicate stronger reconstruction capability.

### Membership inference attack

A Membership Inference Attack (MIA) aims to determine whether a specific record $\left(x^{⋆},y^{⋆}\right)$ was used in the training dataset of the target model $f_{\theta }$. We adopt the widely recognized black-box loss-threshold attack of Yeom et al. [48] because it remains one of the strongest and most commonly used baselines, exhibiting minimal assumptions and consistent effectiveness. The attacker, with only query access to the model, computes the per-sample as specified in the Equation 9:


$$\begin{array}{c} \ell (x^{⋆},y^{⋆})=ℒ(f_{\theta }(x^{⋆}),y^{⋆}), \end{array}$$ (9)

where ℒ represents the training loss function. The decision rule is threshold-based: the record is inferred as a member if $\ell (x^{⋆},y^{⋆})<T_{MIA}$. Following Yeom et al., we set the threshold *T*_MIA_ to the median loss over a reference population as in the Equation 10:


$$\begin{array}{c} T_{MIA}=median\{\ell (x_{i},y_{i})\}. \end{array}$$ (10)

This threshold exploits the observation that machine learning models systematically achieve lower loss on training examples than on unseen examples. We measure attack performance using the membership advantage using the Equation 11:


$$\begin{array}{c} {Adv}_{MIA}=TPR-FPR, \end{array}$$ (11)

where TPR is the true positive rate and FPR is the false positive rate. This metric ranges from 0 to 1 and serves as the de facto standard in privacy literature [49–52] due to its robustness and direct interpretability.

${Adv}_{MIA}\leq 0.05$ indicates negligible risk, while values above 0.10 show significant privacy leakage. For comparative interpretation, we define an MIA resistance score as


$$\begin{array}{c} R_{MIA}=1-{Adv}_{MIA}, \end{array}$$

where higher values indicate stronger resistance to membership inference.

### Malicious client attack (label-flipping poisoning)

To evaluate robustness against adversarial behavior, we simulate label-flipping attacks in which malicious clients deliberately corrupt training labels [53–55]. For binary classification, labels are inverted as shown in Equation 12:


$$\begin{array}{c} y_{i}^{mal}=1-y_{i}. \end{array}$$ (12)

The corrupted labels generate malicious gradients as specified in Equation 13:


$$\begin{array}{c} {\tilde{g}}_{i}=\nabla_{\theta }ℒ(f_{\theta }(x_{i}),y_{i}^{mal}), \end{array}$$ (13)

These corrupted updates skew the global model towards inverted class labels, leading to substantial degradation of overall performance. To thoroughly evaluate robustness against label-flipping attacks, we monitor the Area Under the Curve (AUC) trend across training rounds with a set of poisoning-resilience metrics (shown in Table 3) with the following parameters that quantify the model’s resistance to label-flipping attacks and its ability to recover after the attack.


$$\begin{array}{c} \begin{array}{cccc} t_{s} & :\text{attack start round}, & t_{e} & :\text{attack end round},\\ T & :\text{final training round}, & \epsilon & :\text{tolerance margin for recovery}. \end{array} \end{array}$$

**Table 3:** Malicious attack evaluation metrics.

Metric	Formula
AUC Before Attack	${AUC}_{before}=\frac{1}{t_{s}-1}\sum_{t=1}^{t_{s}-1}{AUC}_{t}$
AUC During Attack	${AUC}_{attack}=\frac{1}{t_{e}-t_{s}+1}\sum_{t=t_{s}}^{t_{e}}{AUC}_{t}$
Minimum AUC	${AUC}_{\min}=\min_{t_{s}\leq t\leq t_{e}}{AUC}_{t}$
Recovery Round	$t_{r}=\min_{t>t_{e}}\left\{t\;|\;{AUC}_{t}\geq {AUC}_{before}-\epsilon \right\}$
Attack-Onset Recovery Duration (Primary)	${NR}_{start}=t_{r}-t_{s}$
Post-Attack Recovery Delay (Complementary)	${NR}_{end}=t_{r}-t_{e}$
AUC After Recovery	${AUC}_{after}=\frac{1}{T-t_{r}}\sum_{t=t_{r}+1}^{T}{AUC}_{t}$
AUC Drop	$\Delta_{drop}={AUC}_{before}-{AUC}_{\min}$
Recovery Gain	$\Delta_{recover}={AUC}_{after}-{AUC}_{\min}$
Recovery Ratio	$RecoveryRatio=\frac{\Delta_{recover}}{\Delta_{drop}}$

## Proposed methodology

We propose a unified BCFL-DAI-PUB framework that integrates blockchain governance, dynamic adaptive incentives, and differential privacy to enable a novel privacy–utility balanced credit-risk assessment model. Further, the framework proves robustness against both adversarial manipulations and inference-based attacks. The framework facilitates secure and decentralized collaboration across financial institutions through smart contracts that standardize global data representation, enforce optimal feature selection, verify and aggregate client updates, and securely maintain reputation and contribution scores and incentivize clients based on both privacy and utility to reach a privacy–utility trade-off. At the fundamental level of the framework, the Dynamic Adaptive Incentive (DAI) mechanism rigorously assesses the client’s performance in terms of both utility and privacy. This method encourages honest and high-quality participation while diminishing the free-riders and malicious actors, which helps ensure reliable aggregation and better global model performance. In addition, adding optimizer-level differential privacy provides formal, end-to-end protection against privacy breaches like membership inference and gradient inference attacks

The following subsections including *1) Smart Contract-Driven Global data representation and Feature selection, 2) Global Model Initialization using Transfer Learning, 3) Client model update and peer-to-peer Evaluation, 4) Dynamic incentive allocation using reputation and contribution score, and finally 5) Global weighted aggregation-reputation based* elaborate on each component of the BCFL-DAI-PUB architecture to ensure a secure, efficient, and high-fidelity decentralized financial learning environment.

### Smart contract-driven global data representation and feature selection

This subsection describes two key operations carried out by the smart contract within our framework: *global data representation and global feature selection*. By coordinating these steps, the framework maintains model consistency, reduces redundant information, and supports more stable training with faster convergence and improved predictive performance.

#### Global data representation.

To ensure consistent data representation across participating institutions, the proposed framework relies on a smart contract–controlled preprocessing process. This design helps reduce the inconsistencies commonly found in distributed financial datasets while supporting privacy-preserving model training. The overall process is organized into four verifiable stages, which are described in the following subsection.

a) **Numerical Attribute Processing**
Numerical attribute processing is designed to maintain consistency across clients by computing shared statistical parameters at the global level.
  - *Missing value imputation:* Each client calculates the local mean for every numerical feature and submits these values to the smart contract. The contract then aggregates the local statistics to compute a global mean as shown in Equation 14, which is used to handle missing values consistently across all participants.

$$\begin{array}{c} \mu_{\text{global}}=\frac{1}{N}\sum_{i=1}^{N}\mu_{i} \end{array}$$ (14)

- *Normalization:* clients report their local minimum and maximum values for each numerical attribute. These values are aggregated by the smart contract to arrive at global min–max values, ensuring that numerical attributes are normalized uniformly across all clients.

b) **Categorical Attribute Processing**
A unified categorical attribute representation is required to ensure consistent handling of categorical features across institutions.
  - *Global dictionary formation:* Each client contributes the unique values for its categorical attributes. These locally collected values are then combined by the smart contract to form a single global dictionary as shown in Equation 15,

$$\begin{array}{c} C_{\text{global}}=\overset{N}{\underset{i=1}{⋃}}C_{i} \end{array}$$ (15)

- *One-hot encoding:* The on-chain dictionary serves as the authoritative reference, ensuring identical encoding dimensions across institutions.

c) **Schema Publication**
Once global statistics and categorical dictionaries are finalized, the smart contract compiles them into an official global schema.
  - *Global Schema packaging:* The contract packages $\mu_{\text{global}}$, global Min–Max parameters, and *C*_global_ into a standardized JSON schema stored in InterPlanetary File System (IPFS).
  - *Global Schema Publishing:* Smart Contract immutably records the schema hash on-chain, which ensures: verifiable preprocessing rules, tamper-proof auditability, synchronized updates across all clients
d) **Local Transformation Using the Global Schema:** Clients download the verified schema and apply identical preprocessing steps to their private datasets, thereby ensuring final preprocessing.
  - *Numerical Transformation:* Clients impute missing numerical values using $\mu_{\text{global}}$. They then scale each feature using the globally agreed Min–Max parameters.
  - *Categorical Transformation:* Clients perform One-Hot Encoding using the fixed global ordering of *C*_global_, which guarantees identical categorical representation across all institutions.

Following preprocessing, the initial dataset comprising 122 raw attributes is transformed into a feature space of 245 attributes as a result of categorical encoding.

#### Global feature selection using explainable AI (XAI) with SHAP.

Once global preprocessing is completed, feature selection is carried out using SHAP-based importance scores computed across participating clients. All numerical computations are carried out off-chain, while the blockchain is used only to store verifiable references to the results. The feature selection procedure consists of two stages.

a) **Local feature importance computation:** Each client independently trains a LightGBM model using its locally preprocessed dataset and evaluates feature importance using SHAP values. For a feature *f*, the importance score at client *i* is defined as the average of the absolute SHAP values over all samples as shown in Equation 16.

$$\begin{array}{c} {ℐ}_{i}(f)=\frac{1}{n_{\text{samples}}}\sum_{j=1}^{n_{\text{samples}}}|\phi_{j}(f)| \end{array}$$ (16)

where $\phi_{j}(f)$ represents the SHAP contribution associated with feature *f* for sample *j*. The computed importance scores are retained outside the blockchain environment. For verification purposes, each client records a cryptographic hash of the importance scores on the smart contract.

b) **Global aggregation and feature selection:** After receiving all client submissions, the global importance of each feature is computed by averaging the corresponding local importance values as represented in Equation 17.

$$\begin{array}{c} {ℐ}_{\text{global}}(f)=\frac{1}{N}\sum_{i=1}^{N}{ℐ}_{i}(f) \end{array}$$ (17)

Features are first ordered based on their global importance values ${ℐ}_{\text{global}}(f)$. The ordered features are examined in decreasing order of importance, and their contributions are accumulated until the cumulative total accounts for 75% of the overall importance. The features included within this threshold constitute the final selected set. To preserve traceability, the identifiers of the selected features, along with a cryptographic hash of the aggregated importance scores, are stored on the blockchain.

Using this procedure, 184 features are selected from the original set of 245 preprocessed attributes. Restricting subsequent training rounds to this reduced feature set lowers communication overhead and simplifies model training. The overall workflow for global preprocessing and feature selection is summarized in Algorithm 1.

**Algorithm 1 Global Data Representation and Global Feature Selection**

**Require:**: Number of clients *N*, cumulative importance threshold *T*_FS_ = 0.75.

**Ensure:** Preprocessed optimal global feature set ${ℱ}_{\text{final}}$

**// Phase A: Global Data Representation**

1: **for** each client *i* = 1 **to** *N* **in parallel do**

2:  $\mu_{i}\leftarrow {\text{Client}}_{i}.\text{ComputeLocalMean}()$

3:  $\min_{i},\max_{i}\leftarrow {\text{Client}}_{i}.\text{ComputeLocalMinMax}()$

4:  ${𝒞}_{i}\leftarrow {\text{Client}}_{i}.\text{ExtractLocalCategories}()$

5:  $\text{SC}\leftarrow \text{submitLocalStats}(\mu_{i},\min_{i},\max_{i})$

6:  $\text{SC}\leftarrow \text{SC}.\text{submitCategories}({𝒞}_{i})$

7: **end for**

8: $\mu_{\text{global}}\leftarrow \frac{1}{N}\sum_{i=1}^{N}\mu_{i}$

9: GlobalMinMaxParams←SC.AggregateMinMax()

10: ${𝒞}_{\text{global}}\leftarrow {⋃}_{i=1}^{N}{𝒞}_{i}$ ▷ Unified global vocabulary

11: $\text{schema}\leftarrow \{\mu_{\text{global}},\text{GlobalMinMaxParams},{𝒞}_{\text{global}}\}$

12: cid←UploadToIPFS(schema)

13: SC.publishGlobalConfig(cid)

14: **for** *i* = 1 **to** *N* **do**

15:  (μ,MinMax,𝒞)←FetchFromIPFS(cid)

16:  ${𝐗}_{i}^{\prime }\leftarrow ImputeMissing({𝐗}_{i},\mu )$

17:  ${𝐗}_{i}^{\prime }\leftarrow MinMaxScale({𝐗}_{i}^{\prime },MinMax)$

18:  ${𝐗}_{i}^{\prime }\leftarrow OneHotEncode({𝐗}_{i}^{\prime },𝒞)$

19: **end for**

**// Phase B: Global Feature Selection via SHAP**

20: **for** each client *i* = 1 **to** *N* **in parallel do**

21:  $M_{i}\leftarrow \text{LightGBM}({𝐗}_{i}^{\prime })$

22:  $I_{i}(f)\leftarrow \text{TreeExplainer}(M_{i}).\text{mean\_abs\_shap\_values()}\;\forall f$

23:  $\text{SC}\leftarrow \text{submitFeatureScores}(i,I_{i})$

24: **end for**

25: $I_{\text{global}}(f)\leftarrow \frac{1}{N}\sum_{i=1}^{N}I_{i}(f)\;\forall f$

26: Sort features descending by *I*_global_(*f*)

27: Initialize cumulative←0, ${ℱ}_{\text{final}}\leftarrow \emptyset$

28: $\text{TotalImportance}\leftarrow \sum_{f^{\prime }}I_{\text{global}}(f^{\prime })$

29: **for** each feature *f* in sorted order **do**

30:  $\text{cumulative}\leftarrow \text{cumulative}+I_{\text{global}}(f)$

31:  ${ℱ}_{\text{final}}\leftarrow {ℱ}_{\text{final}}\cup \{f\}$

32:  **if** cumulative≥0.75·TotalImportance **then**

33:   **break**

34:  **end if**

35: **end for**

36: $\text{SC}.\text{finalizeFeatureSet}({ℱ}_{\text{final}})$

37: **return** ${ℱ}_{\text{final}}$ ▷ Top 184 features selected

### Global model initialization using transfer learning

Once global preprocessing and feature selection are completed, resulting in a preprocessed set of the top 184 features, the global model is initialized using a two-phase training pipeline such as *1) Phase I: Self-Supervised Transfer Learning for Tabular Representations, and 2) Phase II: Global Diffusion Initialization with DP-Enhanced Optimization*, helps stabilize convergence while strengthening privacy protection during early training.

#### Phase I: Self-supervised transfer learning for tabular representations.

The initialization pipeline begins with a deep tabular transformer serving as the feature extraction backbone. Rather than using random weights, the backbone is pretrained using a self-supervised contrastive objective that maximizes agreement between augmented views of the same input sample without relying on target labels. This pretraining yields invariant and semantically meaningful feature representations.

The learned parameters are transferred to the global model and the backbone remains frozen for the first ten diffusion training rounds to preserve stability under differential privacy noise. Following this pre-training, the backbone is unfrozen and jointly fine-tuned, enabling the network to adapt its representations while preserving privacy-aware robustness.

#### Phase II: Global diffusion initialization with DP-enhanced optimization.

Building on the structured latent space produced by SSL transfer learning, the diffusion model is initialized using the latent embeddings rather than random noise, forming a clean starting state *x*_0_ (see Algorithm 2). To support mixed-type financial data, the framework adopts a *TabDDPM* architecture with specialized forward and reverse processes.

a) **Hybrid Forward Diffusion Process:** The forward process gradually perturbs the latent representation using a predefined variance schedule $\{\beta_{t}{\}}_{t=1}^{T}$. Two parallel noise streams handle heterogeneous features.
  - *Numerical track:* Continuous features are corrupted using Gaussian noise. At any timestep *t*, the system computes the noisy sample $x_{t}$ using the transition rule as specified in Equation 18:

$$\begin{array}{c} x_{t}=\sqrt{{\bar{\alpha }}_{t}}\;x_{0}+\sqrt{1-{\bar{\alpha }}_{t}}\;\epsilon ,\;\epsilon \sim 𝒩(0,I) \end{array}$$ (18)

where $\alpha_{t}=1-\beta_{t}$ and ${\bar{\alpha }}_{t}=\prod_{i=1}^{t}\alpha_{i}$.

- *Categorical track:* Discrete attributes with *K* categories follow a multinomial diffusion rule (as shown in Equation 19:

$$\begin{array}{c} q(c_{t}|c_{t-1})=(1-\beta_{t})𝐯(c_{t-1})+\beta_{t}/K \end{array}$$ (19)

where $𝐯(c_{t-1})$ denotes the one-hot encoding of the previous state.

b) **Reverse Denoising Process:** The reverse process learns to reconstruct the clean data by approximating the posterior distributions. The denoising network $f_{\theta }$ simultaneously predicts the Gaussian noise component for numerical features and the category probabilities for discrete ones. The framework optimizes these parameters by minimizing a joint multi-task loss function (as shown in Equations from 20–22):

$$\begin{array}{c} {ℒ}_{\text{total}}={ℒ}_{\text{mse}}+{ℒ}_{\text{vlb}} \end{array}$$ (20)

The numerical loss (${ℒ}_{\text{mse}}$) matches the predicted noise by calculating the Mean Squared Error between the added noise ϵ and the model’s prediction $\epsilon_{\theta }(x_{t},t)$:

$$\begin{array}{c} {ℒ}_{\text{mse}}={𝔼}_{x_{0},\epsilon ,t}\left[\|\epsilon -\epsilon_{\theta }(x_{t},t){\|}^{2}\right] \end{array}$$ (21)

The categorical loss (${ℒ}_{\text{vlb}}$) employs the Variational Lower Bound, calculated as the KL divergence between the true posterior *q* and the learned reverse transition $p_{\theta }$:

$$\begin{array}{c} {ℒ}_{\text{vlb}}={𝔼}_{q}\left[\sum_{t=1}^{T}D_{KL}(q(c_{t-1}|c_{t},c_{0})\|p_{\theta }(c_{t-1}|c_{t}))\right] \end{array}$$ (22)

c) **Differential Privacy at the Optimizer Layer:** To ensure the diffusion model does not memorize sensitive client records, we incorporate Differential Privacy (DP) at the optimizer layer. After computing per-sample gradients for the joint loss, the system clips their norms to a bound *C* and injects calibrated Gaussian noise before aggregation as shown in Equation 23:

$$\begin{array}{c} \tilde{g}=\frac{1}{N}\sum_{i=1}^{N}\text{clip}(g_{i},C)+𝒩(0,\sigma^{2}C^{2}I) \end{array}$$ (23)

where $g_{i}$ is the gradient of the *i*-th sample, σ is the noise multiplier, and *N* is the batch size.

**Algorithm 2 Global model initialization via SSL transfer learning and TabDDPM with differential privacy**

**Require:** Preprocessed global feature set ℱ, input data **D**, diffusion steps *T*, clipping norm *C*, noise scale σ, unfreeze round *R*_stable_ = 10

**Ensure:** Private global model ${𝐌}_{G}$

**// Phase I: SSL Transfer Learning and Feature Extraction**

1: ${𝐌}_{backbone}\leftarrow InitTabularTransformer(ℱ)$

2: ${𝐖}_{SSL}\leftarrow ContrastivePretrain({𝐌}_{backbone},𝐃)$

3: **Freeze M**_backbone_ layers with weights **W**_SSL_

4: $x_{0},c_{0}\leftarrow {𝐌}_{backbone}(𝐃)$▷ Stable latent representations

**// Phase II: DP-Enhanced Tabular Diffusion (TabDDPM)**

5: ${𝐌}_{diff}\leftarrow InitializeDenoisingNetwork()$

6: ${𝐌}_{G}\leftarrow {𝐌}_{backbone}⊕{𝐌}_{diff}$

7: **for** each training iteration *i* **do**

8:  **if** *i* = *R*_stable_ **then**

9:   **Unfreeze M**_backbone_▷ Privacy-aware fine-tuning

10:  **end if**

11:  Sample timestep t~Uniform{1,...,T}

**// Hybrid forward diffusion**

12:  ϵ~𝒩(0,𝐈)

13:  $x_{t}\leftarrow \sqrt{{\bar{\alpha }}_{t}}x_{0}+\sqrt{1-{\bar{\alpha }}_{t}}\;\epsilon$

14:  $q(c_{t}|c_{t-1})\leftarrow (1-\beta_{t})𝐯(c_{t-1})+\beta_{t}/K$

**// Multi-task denoising and loss**

15:  $\hat{\epsilon },\hat{p}\leftarrow {𝐌}_{G}(x_{t},c_{t},t)$

16:  ${ℒ}_{mse}\leftarrow \|\epsilon -\hat{\epsilon }{\|}^{2}$

17:  ${ℒ}_{vlb}\leftarrow D_{KL}(q(c_{t-1}\;|c_{t},c_{0},\|\;\hat{p})$

18:  ${ℒ}_{total}\leftarrow {ℒ}_{mse}+{ℒ}_{vlb}$

**// DP-optimizer layer**

19:  $g\leftarrow \nabla_{\theta }{ℒ}_{total}$

20:  $\tilde{g}\leftarrow g/\max(1,\|g{\|}_{2}/C)+𝒩(0,\sigma^{2}C^{2}𝐈)$

21:  $\theta \leftarrow \theta -\eta \tilde{g}$

22: **end for**

**return** ${𝐌}_{G}$

The framework achieves stable generative learning and strong privacy protection by combining diffusion-based denoising with differentially private optimization. This layered defense enhances resistance to inference-based attacks, such as gradient leakage and membership inference, while ensuring consistent model behavior across distributed participants.

### Client model update and peer-to-peer evaluation

Once the Global Diffusion Model is initialized using Self-Supervised Learning (SSL) and Diffusion Model, a global model copy is shared to all participating clients, initiating an iterative training process of two auditable phases like *Model Update & Self-Assessment Submission*, and *Peer-to-Peer Mutual Evaluation*

#### Model update & self-assessment submission.

Each client locally trains the received model, computes performance and privacy metrics, and submits the signed results to the blockchain.

a) **Local Model Update:** For client *i* at training round *t*, the local model parameters are updated using standard gradient descent by minimizing the local loss function $L_{i}$ as in Equation 24:

$$\begin{array}{c} {𝐰}_{i,t+1}={𝐰}_{i,t}-\eta \nabla L_{i}({𝐰}_{i,t}), \end{array}$$ (24)

producing the updated local model ${𝐰}_{i,t+1}$.
b) **Utility Metric — Area Under the Curve (AUC):** Predictive utility is quantified using the Area Under the ROC Curve (AUC). The AUC is computed by trapezoidal integration of the ROC curve as shown in Equation 25:

$$\begin{array}{c} \text{AUC}=\sum_{k=1}^{n}\left[({\text{FPR}}_{k+1}-{\text{FPR}}_{k})\frac{{\text{TPR}}_{k}+{\text{TPR}}_{k+1}}{2}\right]. \end{array}$$ (25)

**c) Privacy Metric — Membership Inference Attack (MIA) Advantage:** Privacy leakage is measured through the MIA Advantage, which estimates an adversary’s ability to distinguish training members from non-members using the Equation 26:

$$\begin{array}{c} {Adv}_{MIA}=TPR-FPR, \end{array}$$ (26)

d) **Broadcast and Finalization:** Client *i* packages the model update and self-assessment metrics into a transaction ${𝒯}_{w}$ and broadcasts it to the blockchain network. Validator nodes execute the Practical Byzantine Fault Tolerance (PBFT) consensus protocol to verify authenticity. Upon consensus, the transaction is immutably recorded in a newly created block.

#### Peer-to-peer mutual evaluation.

After all model updates and self-reported metrics are finalized on the ledger, clients collaboratively validate the quality of each submitted update through mutual evaluation.

a) **Model Retrieval:** Each participant retrieves the verified local model updates ${𝐰}_{i,t+1}$ directly from the blockchain.
b) **Computation of Peer Evaluation Scores:** Using its own private data, every evaluator $E_{i}$ assesses all client models by computing two vectors: a Peer Utility Vector and a Peer Privacy Vector for round *t*.
  - **Peer Utility Vector (**${𝐮}_{i}^{t}$**):** This vector (in Equation 27) represents the evaluator $E_{i}$’s AUC-based performance assessment of all *N* client models during round *t*.

$$\begin{array}{c} {𝐮}_{i}^{t}=[u_{i,1}^{t},u_{i,2}^{t},...,u_{i,N}^{t}] \end{array}$$ (27)

- **Peer Privacy Vector (**${𝐩}_{i}^{t}$**):** This vector (in Equation 28) records how evaluator $E_{i}$ quantifies the MIA Advantage (vulnerability) for all *N* local models.

$$\begin{array}{c} {𝐩}_{i}^{t}=[p_{i,1}^{t},p_{i,2}^{t},...,p_{i,N}^{t}] \end{array}$$ (28)

c) **Package the Transaction (**${𝒯}_{u}$**):** Clients package the computed and signed Utility Vector (${𝐮}_{i}^{t}$) and Privacy Vector (${𝐩}_{i}^{t}$) into a single evaluation transaction, ${𝒯}_{u}$.
d) **Submit Evaluation:** Clients broadcast this signed transaction package (${𝒯}_{u}$). A subsequent consensus round then verifies and records this evaluation block on the blockchain, making the peer evaluation results available for the subsequent steps of reputation score and contribution score calculation.

Algorithm 3 defines the entire decentralized workflow, outlining the initial sharing of the local SSL-based Diffusion Model (integrated with DP at the optimizer), local model training, subsequent metric assessment, and final peer-to-peer evaluation.

**Algorithm 3 Model update and peer-to-peer evaluation**

**Require:** ${𝐰}_{i,t}$ (current model weights), ${𝒟}_{i}$ (local client data), η (learning rate), *N* (number of clients)

**Ensure:** ${𝒯}_{u}$ (verified peer evaluation transaction recorded on the blockchain)

**Phase A: Model contribution and verification**

1: ${𝐰}_{i,t+1}\leftarrow {𝐰}_{i,t}-\eta \nabla L_{i}({𝐰}_{i,t})$

2: ${\text{AUC}}_{i}\leftarrow \text{ComputeAUC}({𝐰}_{i,t+1},{𝒟}_{i})$

3: ${\text{MIA}}_{\text{Adv},i}\leftarrow \text{ComputeMIA}({𝐰}_{i,t+1},{𝒟}_{i})$

**// Secure on-chain recording**

4: ${𝒯}_{w}\leftarrow \text{PackageUpdate}({𝐰}_{i,t+1},{\text{AUC}}_{i},{\text{MIA}}_{\text{Adv},i})$

5: ${\text{Client}}_{i}.\text{Broadcast}({𝒯}_{w})$

6: **repeat**

7:  $\text{Verified}\leftarrow \text{Validators.ExecutePBFT}({𝒯}_{w})$

8: **until** *Verified* = True

9: $\text{Blockchain.RecordBlockAssembly}({𝒯}_{w})$

**Phase B: Peer-to-peer mutual evaluation**

10: ${𝐰}_{\text{all},t+1}\leftarrow {\text{Client}}_{i}.\text{DownloadVerifiedModels}()$

11: $u_{i}^{t}\leftarrow {\text{Client}}_{i}.\text{ComputePeerUtilityScores}({𝐰}_{\text{all},t+1},{𝒟}_{i})$

12: $p_{i}^{t}\leftarrow {\text{Client}}_{i}.\text{ComputePeerPrivacyScores}({𝐰}_{\text{all},t+1},{𝒟}_{i})$

13: ${𝒯}_{u}\leftarrow \text{PackageVectors}(u_{i}^{t},p_{i}^{t})$

14: ${\text{Client}}_{i}.\text{Broadcast}({𝒯}_{u})$

15: **repeat**

16:  $\text{Verified}\leftarrow \text{Validators.ExecutePBFT}({𝒯}_{u})$

17: **until** *Verified* = True

18: $\text{Blockchain.RecordEvaluationBlock}({𝒯}_{u})$

**return** ${𝒯}_{u}$

### Dynamic incentive allocation using reputation and contribution scores

Peer evaluation results (${𝐮}_{i}^{t}$ and ${𝐩}_{i}^{t}$), which are permanently recorded on the blockchain, enable the Smart Contract (SC) to compute each client’s reputation and activate the Dynamic Adaptive Incentive mechanism. This mechanism systematically regulates participant behavior to sustain an optimal balance between predictive utility and privacy preservation in two stages, like *Reputation System Design & Scoring*, and *Contribution-Incentive Mapping*

#### Reputation system design & scoring.

At the end of each communication round, the smart contract automatically computes and updates each client’s reputation score through the following sequential steps:

a) **Score Aggregation:** The SC first reads all peer evaluation transactions (${𝒯}_{u}$) and calculates the Average Utility (${\overset{―}{U}}_{i^{\prime }}^{t}$) and Average Privacy (${\overset{―}{P}}_{i^{\prime }}^{t}$) as shown in Equation 29 received by each client $i^{\prime }$ as the mean of all *N* peer evaluation values:

$$\begin{array}{ccc} {\overset{―}{U}}_{i^{\prime }}^{t} & =\frac{1}{N}\sum_{i=1}^{N}u_{i}^{t}[i^{\prime }]\;\text{and}\;{\overset{―}{P}}_{i^{\prime }}^{t} & =\frac{1}{N}\sum_{i=1}^{N}p_{i}^{t}[i^{\prime }] \end{array}$$ (29)

b) **Overall Evaluation.** At each communication round *t*, the smart contract computes the overall utility and privacy reference values by averaging the peer-evaluated scores across all participating clients as shown in Equation 30:

$$\begin{array}{ccc} U^{t} & =\frac{1}{N}\sum_{i=1}^{N}{\overset{―}{U}}_{i}^{t}\;\text{and}\;P^{t} & =\frac{1}{N}\sum_{i=1}^{N}{\overset{―}{P}}_{i}^{t} \end{array}$$ (30)

c) **Normalized Reputation Scores in** [−1,1]**.** Using the round-wise reference values $U^{t}$ and $P^{t}$, client reputations are obtained by mapping deviations from these averages into the interval [−1,1] using a shifted sigmoid function. The utility and privacy reputation score for client *i* at round *t* is defined as in Equation 31.

$$\begin{array}{ccc} {\text{Rep}}_{U_{i}}^{t} & =2\;\sigma \;\left(\gamma \left({\overset{―}{U}}_{i}^{t}-U^{t}\right)\right)-1\;\text{and}\;{\text{Rep}}_{P_{i}}^{t} & =2\;\sigma \;\left(\gamma \left(P^{t}-{\overset{―}{P}}_{i}^{t}\right)\right)-1 \end{array}$$ (31)

where σ(·) denotes the sigmoid function and γ>0 controls the sensitivity of the reputation update.
d) **Historical Reputation Update.** To limit the effect of short-term variations, reputation scores are updated over time using an exponential moving average as represented in Equation 32:

$$\begin{array}{c} {FR}_{U_{i}}^{t}=(1-\lambda ){FR}_{U_{i}}^{t-1}+\lambda \;{\text{Rep}}_{U_{i}}^{t}\;\text{and}\;{FR}_{P_{i}}^{t}=(1-\lambda ){FR}_{P_{i}}^{t-1}+\lambda \;{\text{Rep}}_{P_{i}}^{t} \end{array}$$ (32)

where λ∈(0,1). Since the instantaneous reputation values lie in [−1,1], the historical reputation scores remain bounded within the same interval.
e) **Total Reputation Score.** The overall reputation of client *i* at round *t* is obtained by combining the historical utility and privacy components as specified in Equation 33

$$\begin{array}{c} {\text{TR}}_{i}^{t}={FR}_{U_{i}}^{t}+{FR}_{P_{i}}^{t} \end{array}$$ (33)

#### Contribution–incentive mapping.

This stage translates reputation values into contribution scores, which are then used to determine the incentives assigned to each client.

a) **Computing contribution scores in** [−1,1]**.** Each client’s contribution is evaluated along two dimensions: predictive utility and privacy preservation. The contribution score reflects both the client’s relative data share and its historical reputation, while remaining within the interval [−1,1]. The client’s data share is first mapped linearly to [−1,1] as shown in Equation 34:

$$\begin{array}{c} \pi_{i}=2\frac{\left|D_{i}\right|}{\left|D\right|}-1 \end{array}$$ (34)

The utility and privacy contribution scores are then computed as weighted combinations of the mapped data share and the corresponding historical reputation values as represented in Equation 35:

$$\begin{array}{ccc} UC_{i}(t) & =\alpha_{c}\;\pi_{i}+(1-\alpha_{c},{FR}_{U_{i}}^{t}\;\text{and}\;PC_{i}(t) & =\alpha_{c}\;\pi_{i}+(1-\alpha_{c})\;{FR}_{P_{i}}^{t} \end{array}$$ (35)

where $\alpha_{c}\in [0,1]$ determines the relative influence of data volume and historical behavior. Since both components lie in [−1,1], the resulting contribution scores also remain within this interval.
b) **Executing the Dynamic Adaptive Incentive mechanism:** Based on the contribution values for predictive utility and privacy preservation, the Dynamic Adaptive Incentive (DAI) framework determines the incentives to be allocated to each client. The framework evaluates each client’s performance by computing its model status through a joint assessment of utility $UC_{i}(t)$ and privacy contribution $PC_{i}(t)$. The model status helps to adapt the appropriate incentives to the client. This two-stage process, comprising model-status evaluation and subsequent incentive allocation, is explained as follows:
  - *Model Status Function:* Clients are categorized in one of five contribution classes using the model status function Ψ(·), which assesses the combined behavior of $PC_{i}(t)$ and $UC_{i}(t)$ as defined in Equation 36, with the DAI decision threshold *T*_DAI_ = 0.70:

$$\begin{array}{cc} \Psi (PC_{i}(t),UC_{i}(t)) & =\\ \;\left\{ \begin{array}{cc} \text{Balanced}, & PC_{i}(t)>T_{DAI}\wedge UC_{i}(t)>T_{DAI},\\ {}[2pt]\text{Privacy Heavy}, & PC_{i}(t)>T_{DAI}\wedge 0\leq UC_{i}(t)\leq T_{DAI},\\ {}[2pt]\text{Utility Heavy}, & 0\leq PC_{i}(t)\leq T_{DAI}\wedge UC_{i}(t)>T_{DAI},\\ {}[2pt]\text{Low Contribution}, & 0\leq PC_{i}(t)\leq T_{DAI}\wedge 0\leq UC_{i}(t)\leq T_{DAI},\\ {}[2pt]\text{No Contribution}, & PC_{i}(t)<0\wedge UC_{i}(t)<0. \end{array}\right. \end{array}$$ (36)

- *Incentive Update Rule:* Based on the determined class Ψ(·), the system executes the incentive update rule ${ℐ}_{i}$ applied to client *i*. This rule guides the dynamic adjustment of incentives, which may include differential privacy (DP) adjustments, scaffold incentives [56,57], and monetary benefits, as specified in Equation 37.

$$\begin{array}{c} {ℐ}_{i}=\left\{ \begin{array}{cc} M\leftarrow M+\text{monetary\_benefit}, & \text{if }\Psi =\text{Balanced},\\ S\leftarrow \text{global\_correction\_error}, & \text{if }\Psi =\text{Privacy Heavy},\\ DP\leftarrow DP-0.1, & \text{if }\Psi =\text{Utility Heavy},\\ S\leftarrow \text{global\_correction\_error}, & \text{if }\Psi =\text{Low Contribution},\\ \text{No Incentive}, & \text{if }\Psi =\text{No Contribution}. \end{array}\right. \end{array}$$ (37)

Table 4 presents the complete decision-making logic of the proposed mechanism, clearly demonstrating how the system dynamically intervenes to preserve global stability and enforce a balanced privacy–utility equilibrium across all clients.

**Table 4:** Proposed dynamic adaptive incentives based on contribution scores.

Condition (Ψ)	Model Status	Incentive Rule ($I_{i}$)	Primary Action
$PC_{i}(t)>0.7\wedge UC_{i}(t)>0.7$	Balanced	M←M+Monetary	Reward
$0\leq PC_{i}(t)\leq 0.7\wedge UC_{i}(t)>0.7$	Utility Heavy	DP←DP−0.1	DP-Boost
$PC_{i}(t)>0.7\wedge 0\leq UC_{i}(t)\leq 0.7$	Privacy Heavy	global_correction_error	Scaffold Incentive
$0\leq PC_{i}(t)\leq 0.7\wedge 0\leq UC_{i}(t)\leq 0.7$	Low Contribution	global_correction_error	Scaffold Incentive
$PC_{i}(t)<0\wedge UC_{i}(t)<0$	No Contribution	No Incentive	No Incentive

**Algorithm 4 Smart contract–driven reputation and adaptive incentive mechanism**

**Require:** Number of clients *N*, peer evaluation vectors $\{u_{i}^{t},p_{i}^{t}{\}}_{i=1}^{N}$, client data sizes $\{|D_{i}|{\}}_{i=1}^{N}$, parameters γ>0, λ∈(0,1), $\alpha_{c}\in [0,1)$, threshold *T*_DAI_

**Ensure:** Updated historical reputations $\{{FR}_{U_{i}}^{t},{FR}_{P_{i}}^{t}{\}}_{i=1}^{N}$ and incentives $\{{ℐ}_{i}{\}}_{i=1}^{N}$

**// Phase A: Reputation score calculation**

1: Read all peer evaluation transactions $\left\{{𝒯}_{u}\right\}$

2: **for** each client $i^{\prime }=1$ **to** *N* **do**

3:  ${\overset{―}{U}}_{i^{\prime }}^{t}\leftarrow \frac{1}{N}\sum_{i=1}^{N}u_{i}^{t}[i^{\prime }]$

4:  ${\overset{―}{P}}_{i^{\prime }}^{t}\leftarrow \frac{1}{N}\sum_{i=1}^{N}p_{i}^{t}[i^{\prime }]$

5: **end for**

6: $U^{t}\leftarrow \frac{1}{N}\sum_{i=1}^{N}{\overset{―}{U}}_{i}^{t}$

7: $P^{t}\leftarrow \frac{1}{N}\sum_{i=1}^{N}{\overset{―}{P}}_{i}^{t}$

8: **for** each client *i* = 1 **to** *N* **do**

9:  ${\text{Rep}}_{U_{i}}^{t}\leftarrow 2\;\sigma \;\left(\gamma \left({\overset{―}{U}}_{i}^{t}-U^{t}\right)\right)-1$

10:  ${\text{Rep}}_{P_{i}}^{t}\leftarrow 2\;\sigma \;\left(\gamma \left(P^{t}-{\overset{―}{P}}_{i}^{t}\right)\right)-1$

11:  ${FR}_{U_{i}}^{t}\leftarrow (1-\lambda ){FR}_{U_{i}}^{t-1}+\lambda \;{\text{Rep}}_{U_{i}}^{t}$

12:  ${FR}_{P_{i}}^{t}\leftarrow (1-\lambda ){FR}_{P_{i}}^{t-1}+\lambda \;{\text{Rep}}_{P_{i}}^{t}$

13:  ${\text{TR}}_{i}^{t}\leftarrow {FR}_{U_{i}}^{t}+{FR}_{P_{i}}^{t}$

14: **end for**

**// Phase B: Contribution–incentive mapping**

15: $\left|D|\leftarrow \sum_{j=1}^{N}|D_{j}\right|$

16: **for** each client *i* = 1 **to** *N* **do**

17:  $\pi_{i}\leftarrow 2\frac{\left|D_{i}\right|}{\left|D\right|}-1$

18:  $UC_{i}(t)\leftarrow \alpha_{c}\;\pi_{i}+(1-\alpha_{c},{FR}_{U_{i}}^{t}$

19:  $PC_{i}(t)\leftarrow \alpha_{c}\;\pi_{i}+(1-\alpha_{c},{FR}_{P_{i}}^{t}$

20:  $\Psi (i)\leftarrow \text{ModelStatus}(PC_{i}(t),UC_{i}(t);T_{DAI})$

21:  **if** Ψ(i)=Balanced **then**

22:   M←M+monetary_benefit

23:  **else if** Ψ(i)=Utility Heavy **then**

24:   DP←DP−0.1

25:  **else if** Ψ(i)=Privacy Heavy **then**

26:   S←global_correction_error

27:  **else if** Ψ(i)=Low Contribution **then**

28:   S←global_correction_error

29:  **else if** Ψ(i)=No Contribution **then**

30:   **No Incentive**

31:  **end if**

32: **end for**

**return** $\{{FR}_{U_{i}}^{t},{FR}_{P_{i}}^{t}{\}}_{i=1}^{N}$

### Global weighted aggregation – reputation based

The Reputation-Adjusted Global Aggregation method executes the final model update by using the client’s Total Reputation (${\text{TR}}_{i}$) as the direct weighting factor. The new global model parameters (**w**^(*t*)^) are computed via the reputation-weighted average using the Equation 38:


$$\begin{array}{c} {𝐰}^{\left(t\right)}=\frac{\sum_{i=1}^{N}{\text{TR}}_{i}\cdot {𝐰}_{i}^{\left(t\right)}}{\sum_{i=1}^{N}{\text{TR}}_{i}} \end{array}$$ (38)

This mechanism dynamically skews the aggregation weights based on contribution quality:

- **Weight Suppression (Low Reputation):** For free-riding or malicious clients, ${\text{TR}}_{i}(t)$ is low, which strongly diminishes their influence, neutralizing low-quality updates independent of their dataset size.
- **Weight Preservation (High Reputation):** For honest clients, ${\text{TR}}_{i}(t)$ remains high, allowing their updates to retain appropriate influence during global model aggregation.

By encouraging reliable participants and removing the influence of low-quality contributions, the aggregation mechanism supports more stable convergence and limits the impact of adversarial behavior during training. Algorithm 4 outlines the workflow of dynamic adaptation of incentives based on contribution scores. An overview of the complete framework architecture is depicted in Fig 1.

**Fig 1:**
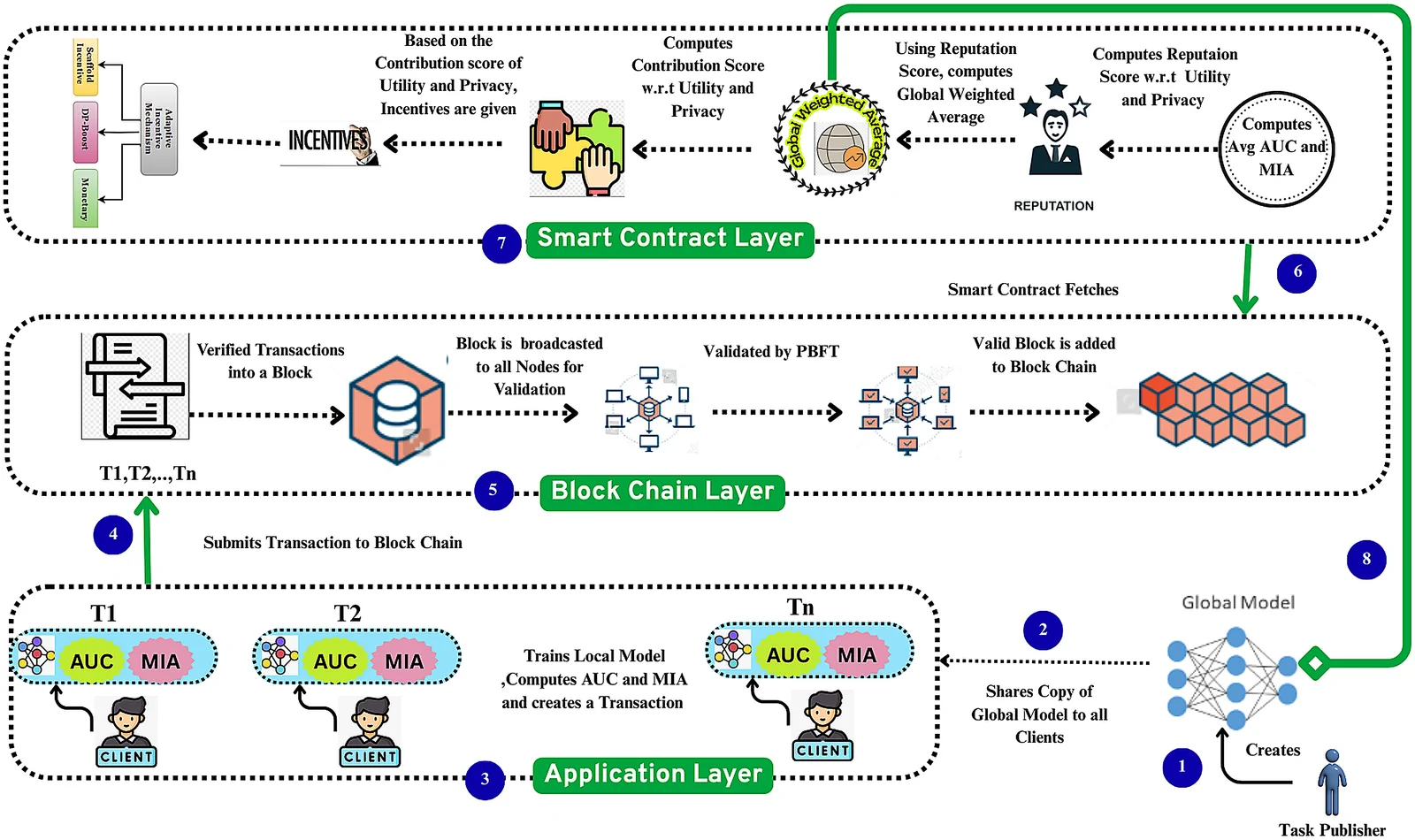
The complete workflow of the proposed BCFL-DAI-PUB framework.

## Results and discussion

The Results and Discussion section follows a structured progression to evaluate BCFL-DAI-PUB under realistic federated learning conditions. We first establish the experimental setting through Dirichlet-based client simulation, which generates both near-IID and non-IID data distributions across five clients. We then show that the proposed framework maintains performance under non-IID data at a level close to that achieved under near-IID conditions. Because real-world federated environments are naturally non-IID, we perform the dynamic adaptive incentive analysis under the non-IID setting. This allows us to examine how the proposed mechanism allocates client-specific incentives to achieve a balance between privacy preservation and utility contribution. We next compare global model performance under both benign and adversarial participation against the BCFL-PS-HER baseline, evaluate resilience to malicious behaviour, membership inference attacks, and gradient inversion attacks, and finally analysed system overhead and throughput. This sequence provides a complete assessment of the proposed framework in terms of effectiveness, privacy, robustness, and deployment feasibility. The Experimental evaluation is organized as:

1. Experimental Setup: Client Simulation
2. Implementation and Reproducibility Settings
3. Parameter Sensitivity Analysis under Different Data Distribution Settings
4. Empirical Analysis of Five Simulated Client Behaviors under the Dynamic Adaptive Incentive (DAI) Mechanism
5. Analysis of Contribution Score Dynamics
6. Comparative Evaluation of Global Model Performance under Honest-Only Participation and Mixed Participation Scenarios (Honest + Malicious + Free-Rider)
7. Assessment of Malicious Attack Resilience and Recovery Efficiency
8. Robustness against Gradient Inversion Attacks (GIA)
9. Protection against Membership Inference Attacks (MIA)
10. System Overhead and Throughput Analysis
11. Predictive Performance Evaluation Using 10-Fold Cross-Validation
12. State-of-the-Art-Comparison

### Experimental setup: Client simulation

We simulated a federated environment with five clients to evaluate the proposed framework under varying participation behaviours and data distribution conditions. The client population comprised three honest clients, one malicious client performing a label-flipping attack, and one free-riding client. This configuration enabled us to examine predictive performance, robustness, incentive adaptation, and overhead efficiency under realistic and non-ideal federated settings.

To study the effect of data heterogeneity, we selected two contrasting Dirichlet concentration values, $\alpha_{D}=0.5$ and $\alpha_{D}=10.0$, representing high and low heterogeneity, respectively. The setting $\alpha_{D}=10.0$ generated a near-IID distribution in which each client retained a class composition close to the global class balance, with only minor variation across clients. As shown in Table 5, the class proportions under this setting remained nearly balanced across all five clients. We therefore used $\alpha_{D}=10.0$ as the near-IID reference case for evaluating the proposed framework under low heterogeneity.

**Table 5:** Client-wise near-IID distribution under Dirichlet concentration parameter $\alpha_{D}=10.0$.

Client	Client Size (%)	Total Records	Class 0	Class 1	Class 0 (%)	Class 1 (%)	Role in Experiment
Client 1	40%	226,149	111,944	114,205	49.50%	50.50%	Honest Contributor
Client 2	25%	141,343	69,258	72,085	49.00%	51.00%	Malicious Actor
Client 3	15%	84,806	42,403	42,403	50.00%	50.00%	Honest Contributor
Client 4	12%	67,844	36,014	31,830	53.08%	46.92%	Honest Contributor
Client 5	8%	45,230	23,067	22,163	51.00%	49.00%	Free-Rider
**Total**	**100%**	**565,372**	**282,686**	**282,686**	–	–	–

In contrast, $\alpha_{D}=0.5$ generated a non-IID distribution with substantially greater client-level heterogeneity. Under this setting, class proportions varied markedly across clients, producing clear label skew in the federated environment. As shown in Table 6, some clients were dominated by class 0, whereas others contained a much larger share of class 1, while the overall global class balance remained preserved. We therefore used $\alpha_{D}=0.5$ as the non-IID setting to assess the stability and robustness of the proposed framework under more realistic heterogeneous conditions.

**Table 6:** Client-wise non-IID distribution under Dirichlet concentration parameter $\alpha_{D}=0.5$.

Client	Client Size (%)	Total Records	Class 0	Class 1	Class 0 (%)	Class 1 (%)	Role in Experiment
Client 1	35%	197,880	138,516	59,364	70.00%	30.00%	Honest Contributor
Client 2	30%	169,612	42,403	127,209	25.00%	75.00%	Malicious Actor
Client 3	20%	113,074	67,844	45,230	60.00%	40.00%	Honest Contributor
Client 4	8%	45,230	24,877	20,353	55.00%	45.00%	Honest Contributor
Client 5	7%	39,576	9,046	30,530	22.86%	77.14%	Free-Rider
**Total**	**100%**	**565,372**	**282,686**	**282,686**	–	–	–

Fig 2 provides a visual comparison of the client-wise class distributions under the two Dirichlet settings. The figure shows that the $\alpha_{D}=10.0$ setting maintained class proportions close to the global balance across clients, whereas the $\alpha_{D}=0.5$ setting introduced clear client-level class imbalance. This contrast highlights the difference between the near-IID and strongly non-IID environments used in the experimental analysis.

**Fig 2:**
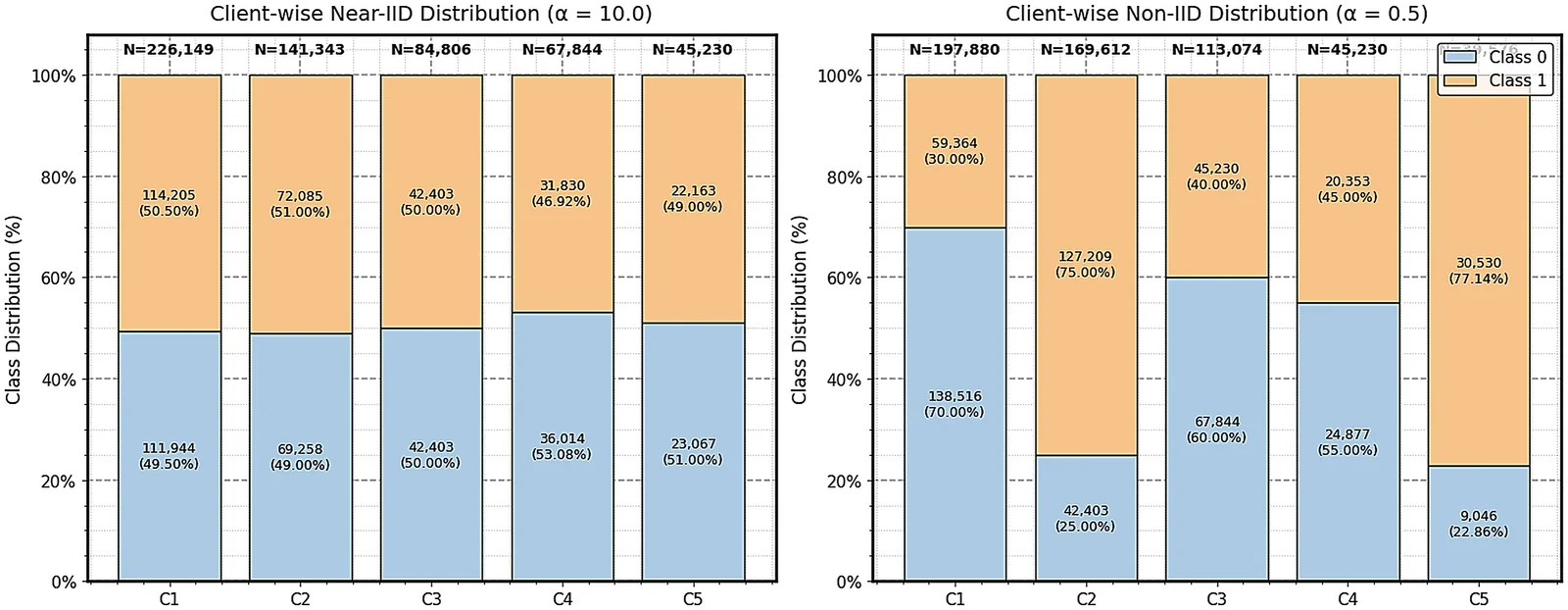
Client-wise class distribution under near-IID ($\alpha_{D}=10.0$) and non-IID ($\alpha_{D}=0.5$) settings.

### Implementation and reproducibility settings

To facilitate reproducibility, the principal implementation parameters used in the Dynamic Adaptive Incentive (DAI) and differential privacy mechanisms are summarized in Table 7.

**Table 7:** Key implementation parameters used in BCFL-DAI-PUB.

Parameter	Value
Reputation sensitivity (γ)	4.0
Historical reputation update weight (λ)	0.30
Contribution weight ($\alpha_{c}$)	0.50
DAI decision threshold (*T*_DAI_)	0.70
DP-Boost privacy-budget adjustment (Δϵ)	0.10
Gradient clipping norm (*C*)	1.0
Gaussian noise multiplier (σ)	0.50
Privacy parameter (δ)	$1\times 10^{-5}$
Initial privacy budget ($\epsilon_{0}$)	1.20
Privacy-budget range ($\epsilon_{\min}$–$\epsilon_{\max}$)	0.40–2.50
Backbone stabilization/unfreeze round (*R*_stable_)	10
Feature-selection cumulative importance threshold (*T*_FS_)	0.75
Number of cross-validation folds	10
Random seed	42

### Parameter sensitivity analysis under different data distribution settings

We evaluated BCFL-DAI-PUB under a near-IID setting ($\alpha_{D}=10.0$) and a strongly non-IID setting ($\alpha_{D}=0.5$) to examine the effect of data heterogeneity on system efficiency. The experimental results for the two Dirichlet concentration settings are presented in Table 8. Compared with the near-IID case, the non-IID setting increased total communication overhead by 6.24% and total memory usage by 3.50%. These results show that stronger client-level heterogeneity introduced only a limited additional system cost. BCFL-DAI-PUB handled skewed client data efficiently, as SCAFFOLD-based correction reduced local update divergence, the incentive mechanism supported reliable participation, and the hybrid coordination design kept communication and storage overhead under control. As a result, the shift from near-IID to non-IID data led to only a marginal increase in overhead.

**Table 8:** Communication and storage overhead under different Dirichlet settings.

Parameter Setting	Communication Overhead(s)					Storage Overhead(MB)			
	Avg.	Avg.	Avg.	Avg.	Avg.	Avg.	Avg.	Avg.	Avg.
	TT	CT	AT	ET	CMT	CNS	CS	MUS	CMS
alpha_10.0	12.56	21.07	1.15	10.25	45.03	4.08	1.69	33.65	39.42
alpha_0.5	13.20	21.58	2.05	11.01	47.84	5.01	1.78	34.01	40.8

*Abbreviations:* TT:Training time; CT: Consensus Time; AT:Aggregation Time; ET:Evaluation time; CMT: Cumulative Communication Time; CNS: Consensus Size; CS: Chain Size; MUS: Model Update Size; CMS: Cumulative Memory size.

We further evaluated predictive performance through 10-fold cross-validation under the same Dirichlet settings. As reported in Table 9, the framework maintained stable performance across both settings, with low variance across the validation folds. Relative to the near-IID setting ($\alpha_{D}=10.0$), the non-IID setting ($\alpha_{D}=0.5$) reduced AUC by 1.10%, Precision by 1.48%, Recall by 0.30%, and F1 Score by 0.86%. Despite these reductions, BCFL-DAI-PUB preserved stable predictive performance under both near-IID and non-IID client distributions, which supports its robustness in practical federated environments.

**Table 9:** 10-fold cross-validation summary under different Dirichlet settings.

Parameter Setting	AUC	Precision	Recall	F1 Score
$\alpha_{D}=10.0$	0.92520±0.00003	0.84992±0.00003	0.75169±0.00008	0.79779±0.00003
$\alpha_{D}=0.5$	0.91501±0.00007	0.83732±0.00116	0.74946±0.00018	0.79096±0.00008

Considering both overhead and predictive performance, the differences between the near-IID and non-IID settings remained small. The non-IID setting introduced only a modest increase in communication and memory overhead, while predictive performance remained stable across both settings. Since practical federated environments are inherently non-IID, we conducted all further experiments on the non-IID distribution. This choice allowed us to evaluate BCFL-DAI-PUB under more realistic conditions.

After analysing the effect of data distribution on overhead and predictive performance, we next turn to client-level behaviour under the Dynamic Adaptive Incentive (DAI) mechanism. Since real-world federated environments are inherently non-IID, the following analysis focuses on the non-IID setting to examine how the proposed mechanism responds to honest, malicious, and free-riding clients.

### Empirical analysis of five simulated client behaviors under the dynamic adaptive incentive (DAI) mechanism

In this section, we evaluate the proposed DAI mechanism across three dimensions. First, we examine DAI-driven behaviour for honest clients towards a privacy–utility balance. Second, we analyse DAI-driven behaviour for the malicious client (C2) and the free-riding client (C5). Finally, we assess the resulting contribution scores to understand how the mechanism differentiates client participation under heterogeneous behaviours.

#### DAI-driven behavior for honest clients toward privacy–utility balance.

The initial stage of the analysis concentrates on the three honest clients shown in Table 5. The results demonstrate that the DAI mechanism consistently directs each cooperative participant toward the intended balanced state, regardless of differences in dataset size or initial contribution conditions. By dynamically adjusting privacy budgets and scaffold corrections, the mechanism incrementally aligns both utility and privacy contributions, leading to stable and reliable convergence at the desired equilibrium.

For the honest clients (C1, C3, and C4), incentives are adjusted during training based on observed utility and privacy contributions. When a client shows high utility with low privacy contribution, privacy protection is strengthened by reducing the privacy budget. When privacy contribution becomes dominant, scaffold-based correction is applied to improve utility and stabilize learning. If both utility and privacy fall below the threshold, the client is treated as low-contributing, and scaffold correction is applied again to encourage effective participation. Once utility and privacy reach a balanced state, monetary incentives are provided to maintain stable behavior. Through these adjustments, all honest clients converge toward a balanced operating state despite differences in data size and initial contribution levels.

Figs 3–5 show that the DAI mechanism intervenes at the very beginning to correct imbalances between privacy and utility for the three honest clients C1, C3, and C4. Through adaptive adjustment of the privacy budget (ε) and activation of SCAFFOLD-based corrections, the framework navigates each participant into synchronized and balanced operating conditions. The experimental results show that all clients converge approximately round 30. This outcome emphasizes, the DAI mechanism’s ability to manage optimizing both privacy and utility automatically, even while having multiple client profiles as participants.

**Fig 3:**
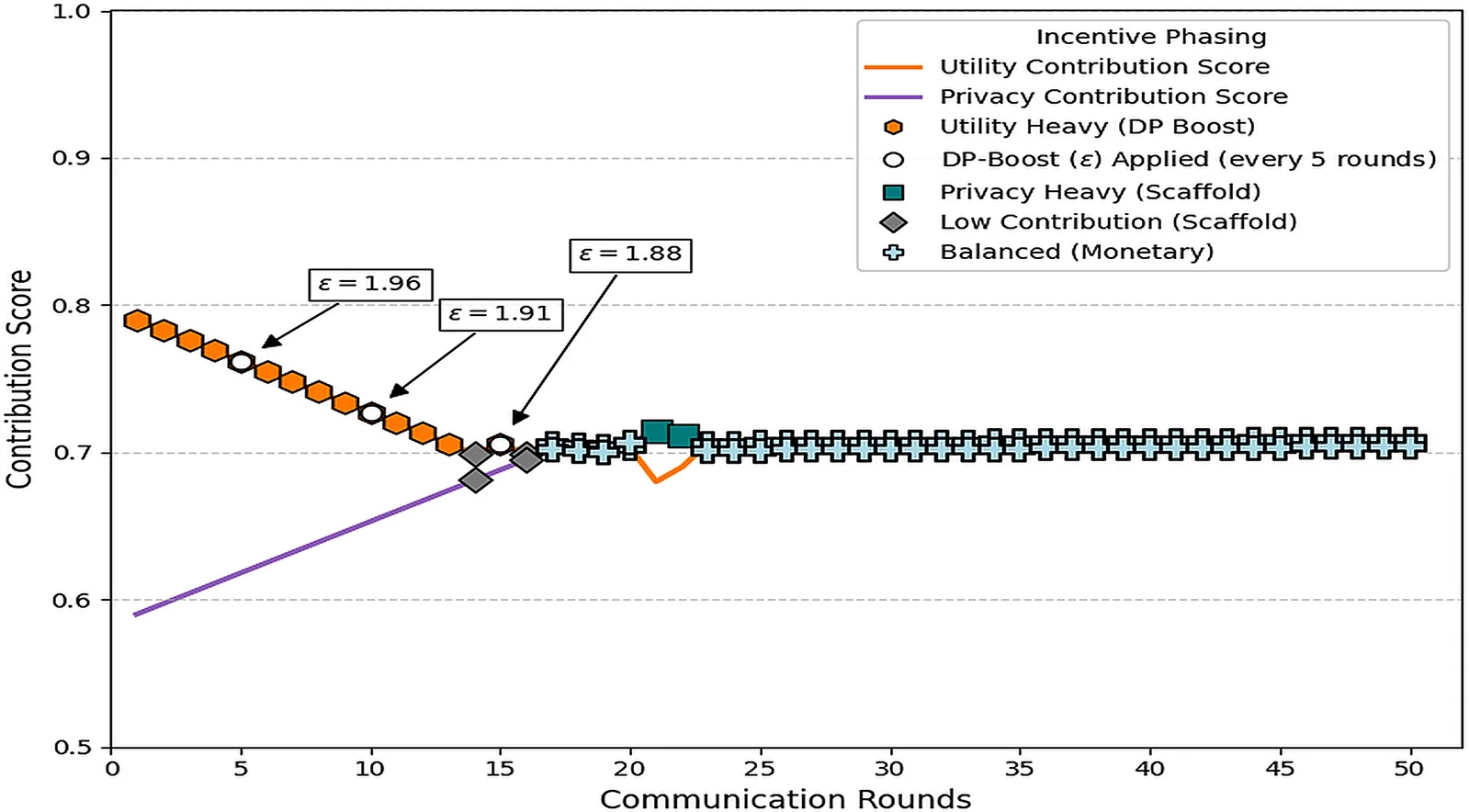
Training stability of C1 toward the optimal privacy–utility trade-off.

**Fig 4:**
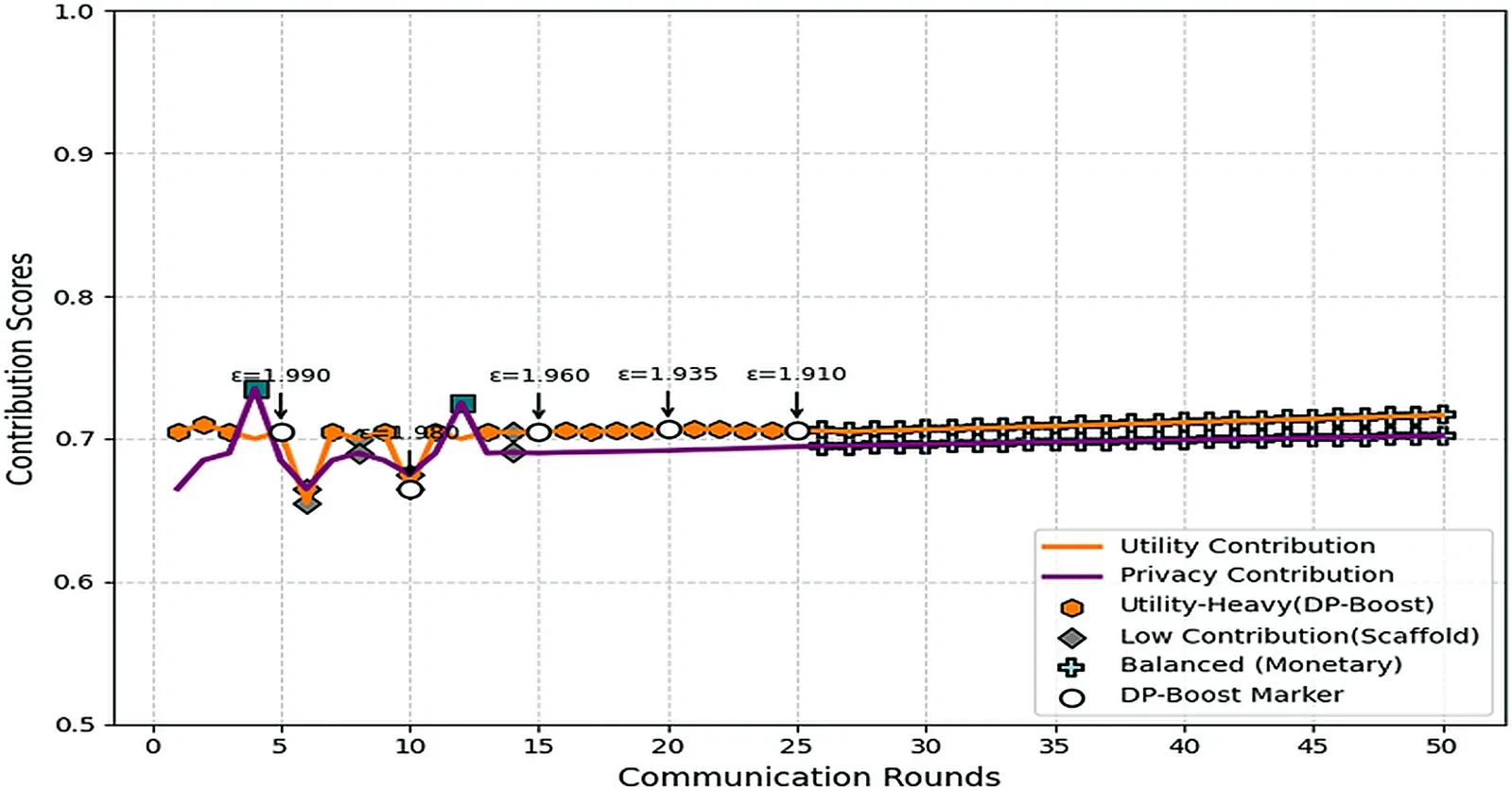
Oscillation damping and convergence behavior of C3.

**Fig 5:**
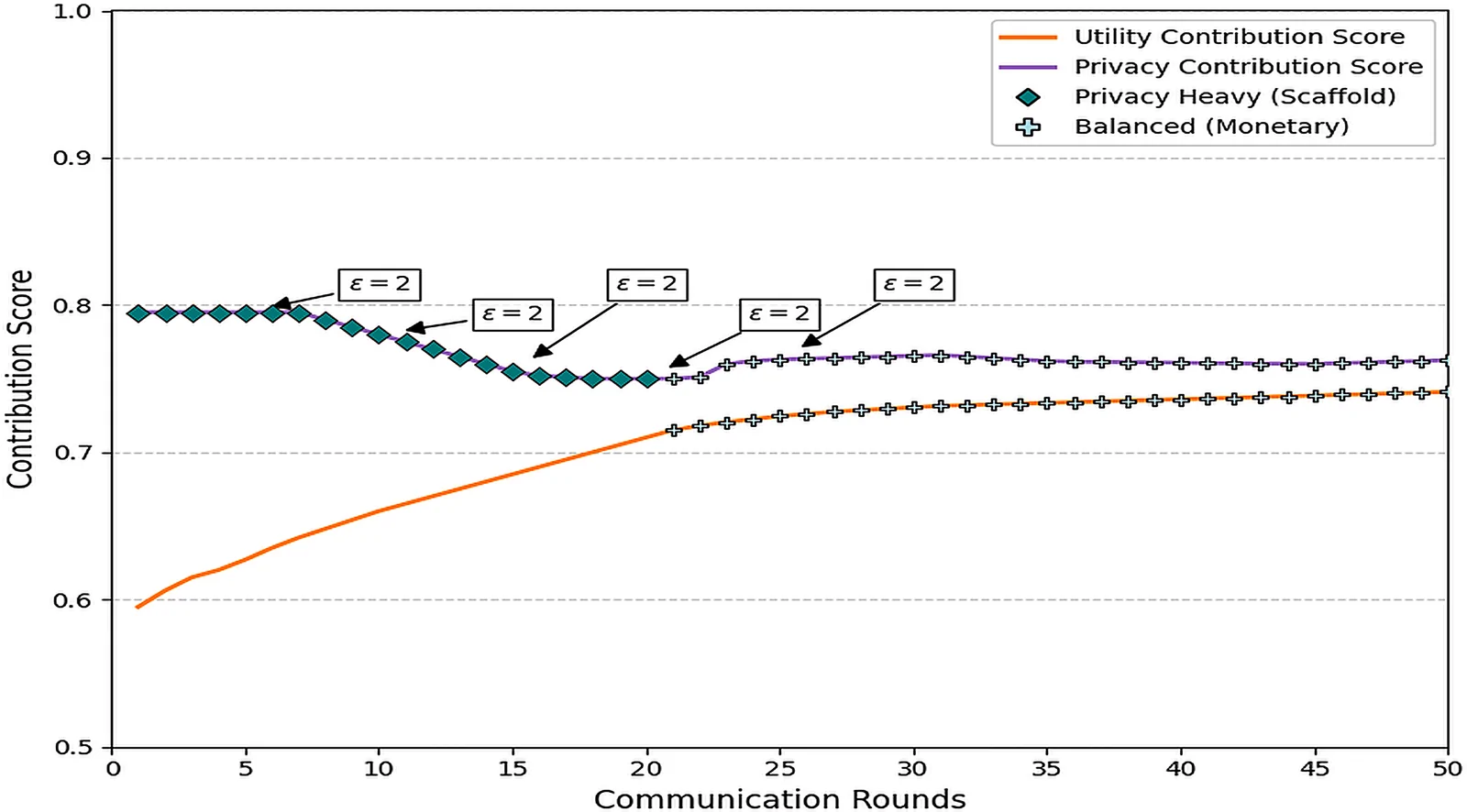
Transition from privacy-heavy to balanced state for Client 4 (small dataset).

#### DAI-driven behavior for malicious Client (C2) and free Rider (C5).

This section describes how the DAI mechanism effectively finds and mitigates the impact of the malicious client (C2) and the free-rider client (C5), by revoking their contribution weights and associated incentives.

Fig 6 describes the behavior of Client C2 during training. In the early rounds, C2 remains in a utility-heavy state, supported by a large local dataset and a privacy budget of ε = 2.0. Up to round 14, it provides strong utility (around 0.85) while maintaining lower privacy values between 0.50 and 0.55, during which privacy protection is gradually strengthened. At round 14, a label-flipping attack is introduced and quickly identified, causing the utility score to fall from 0.78 to −0.25. From round 15 onward, C2 is placed in the no-contribution state, and all incentives are permanently withdrawn.

**Fig 6:**
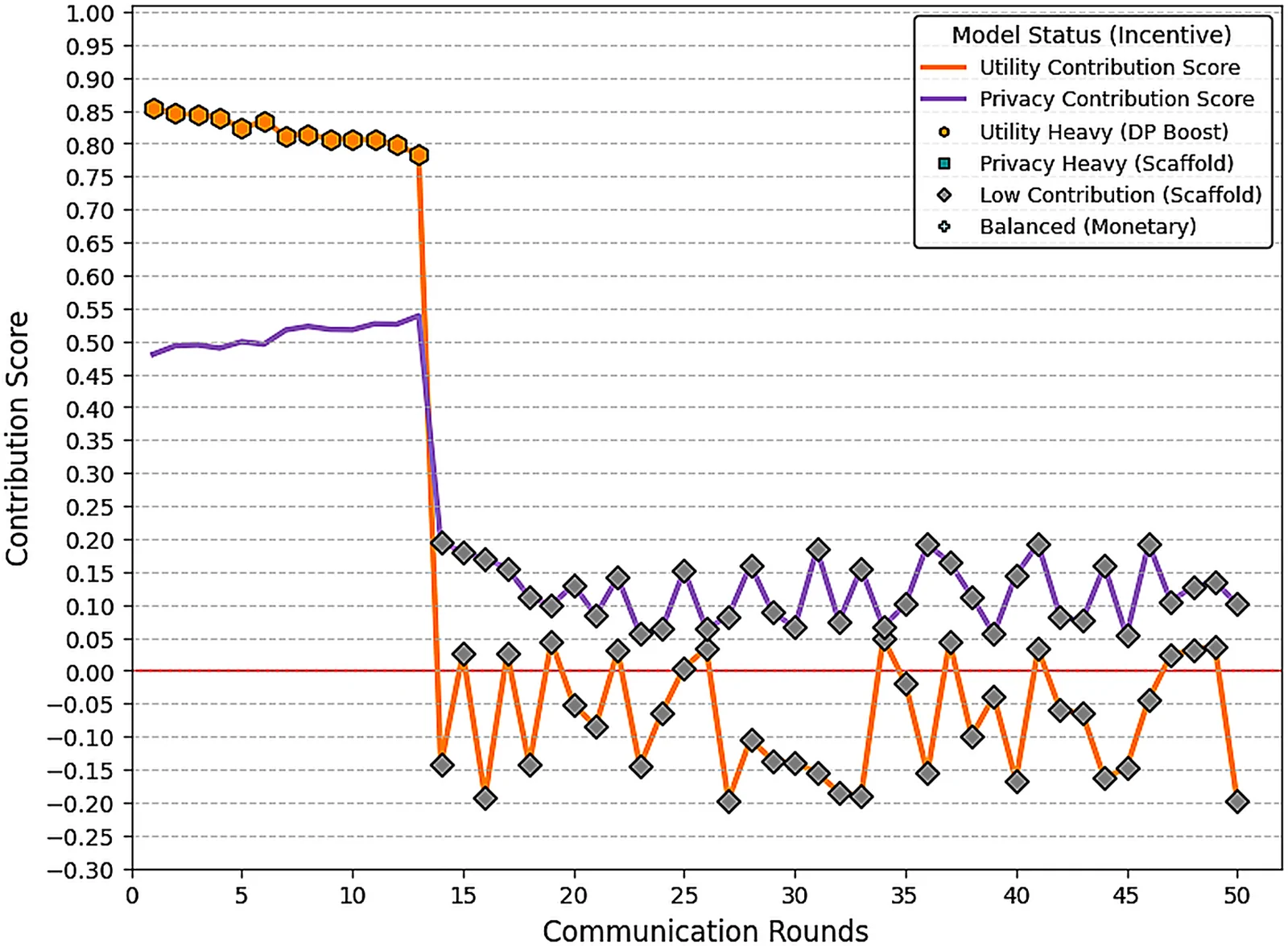
Malicious Client 2—Immediate transition to No-Contribution state and permanent cessation of incentives after label-flipping attack at round 14.

Client C5 shows free-riding behavior from the beginning by submitting outdated updates without meaningful training effort. It operates with a small dataset and a privacy budget of ε = 2.0. Despite repeated corrective actions, its contribution remains consistently low. As a result, C5 is identified as a free rider, moved to the no-contribution state, and excluded from incentives for the remainder of training. Fig 7 reflects its minimal contribution and the continued absence of rewards.

**Fig 7:**
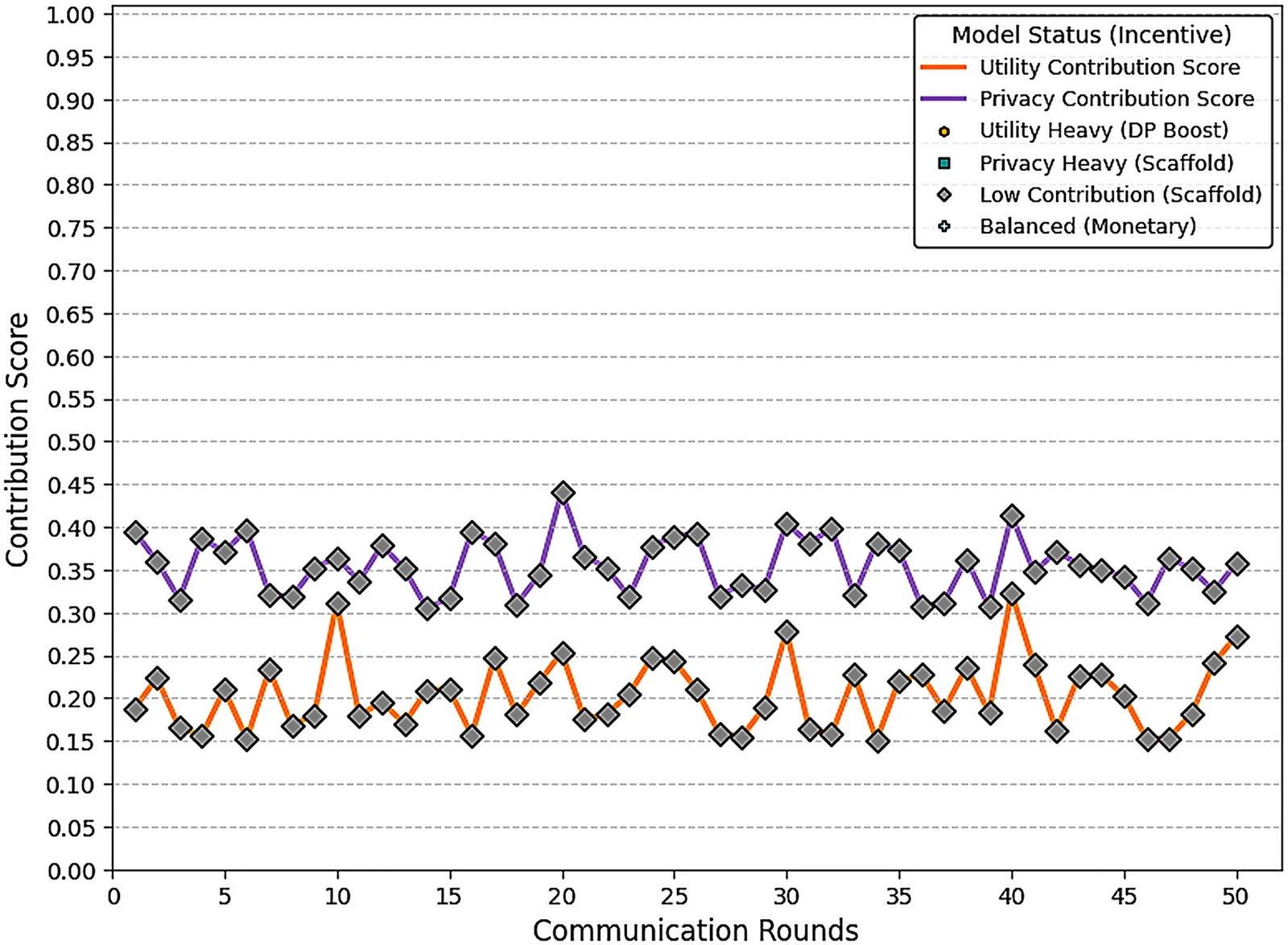
Client 5 (C5)—Free-rider with small dataset.

#### Analysis of contribution score dynamics.

Fig 8 presents the contribution scores of five simulated clients representing different participation behaviors after 50 communication rounds. The scores are summarized based on the threshold of 0.7 as:

- **Honest Clients (C1, C3, C4):** These clients achieve contribution scores between 0.72 and 0.76, all exceeding the threshold of 0.7. Therefore, the system allocates positive incentives that encourage improved global model performance while preserving a utility–privacy equilibrium.
- **Malicious Client (C2):** The malicious client records contribution scores as −0.20, which leads to no incentive allocation and reduced influence on the global model.
- **Free-Rider (C5):** Due to repeated non-contributory updates across training rounds, the free-rider attains a contribution score of 0.35, which is well below the eligibility threshold. As a result, the client receives no incentives and has no influence on the global model performance.

**Fig 8:**
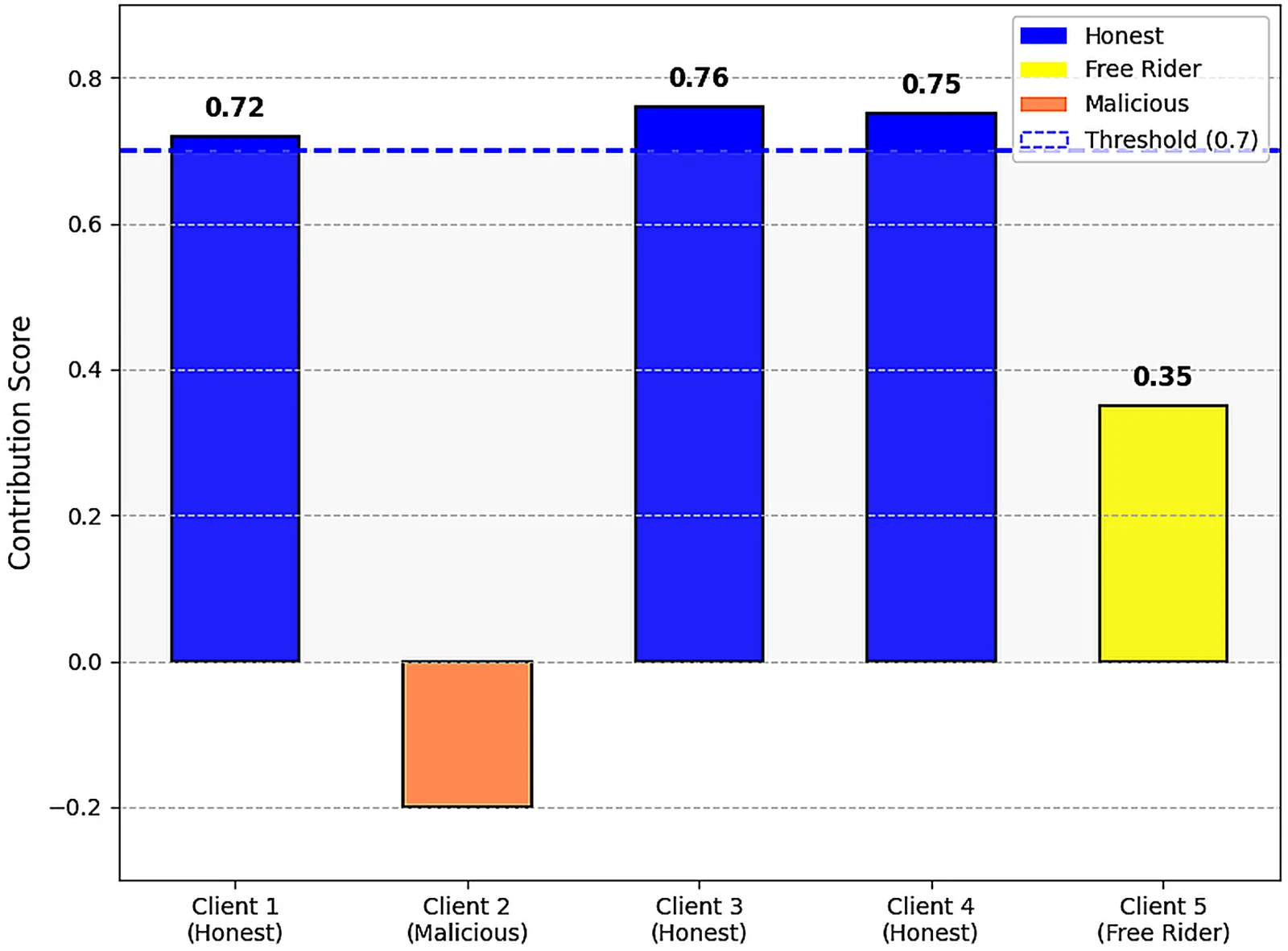
Contribution score of 5 clients (including malicious and free riders).

The above results show that the proposed DAI mechanism rewards honest participation while limiting the influence of malicious and free-riding clients, without disrupting global model performance or the intended privacy–utility balance. Overall, the client-level analysis confirms that the mechanism can differentiate among heterogeneous client behaviours and respond to them appropriately during training. Having established this participant-level effectiveness, we next examine how these behavioural differences affect global model performance under different participation scenarios.

### Comparative evaluation of global model performance under honest-only participation and mixed participation scenarios (Honest + Malicious + Free-Rider)

Building on the client-level results, we next evaluate the end-to-end effectiveness of BCFL-DAI-PUB at the global model level. We assess global model performance across training rounds under two controlled scenarios: *1) Reputation-Aware Optimization in Benign Settings* and *2) Reputation-Aware Robustness Against Malicious Attacks*. These experiments allow us to determine whether dynamic incentive control improves learning quality and robustness under more challenging federated settings. For comparison, we use BCFL-PS-HER, a blockchain-based federated learning baseline that employs static reputation with hand-crafted rules.

#### Reputation-aware optimization in benign settings.

When all clients behave honestly, BCFL-DAI-PUB achieves higher global AUC than BCFL-PS-HER across all communication rounds and reaches a final AUC of 0.93, compared with 0.88 for the baseline (Fig 9). This improvement stems from the proposed framework’s dynamic incentive adjustment and reputation-aware aggregation, which reward consistently useful local updates and assign greater influence to reliable clients during model fusion. These results show that the proposed design improves convergence and overall global model quality even in fully benign settings.

**Fig 9:**
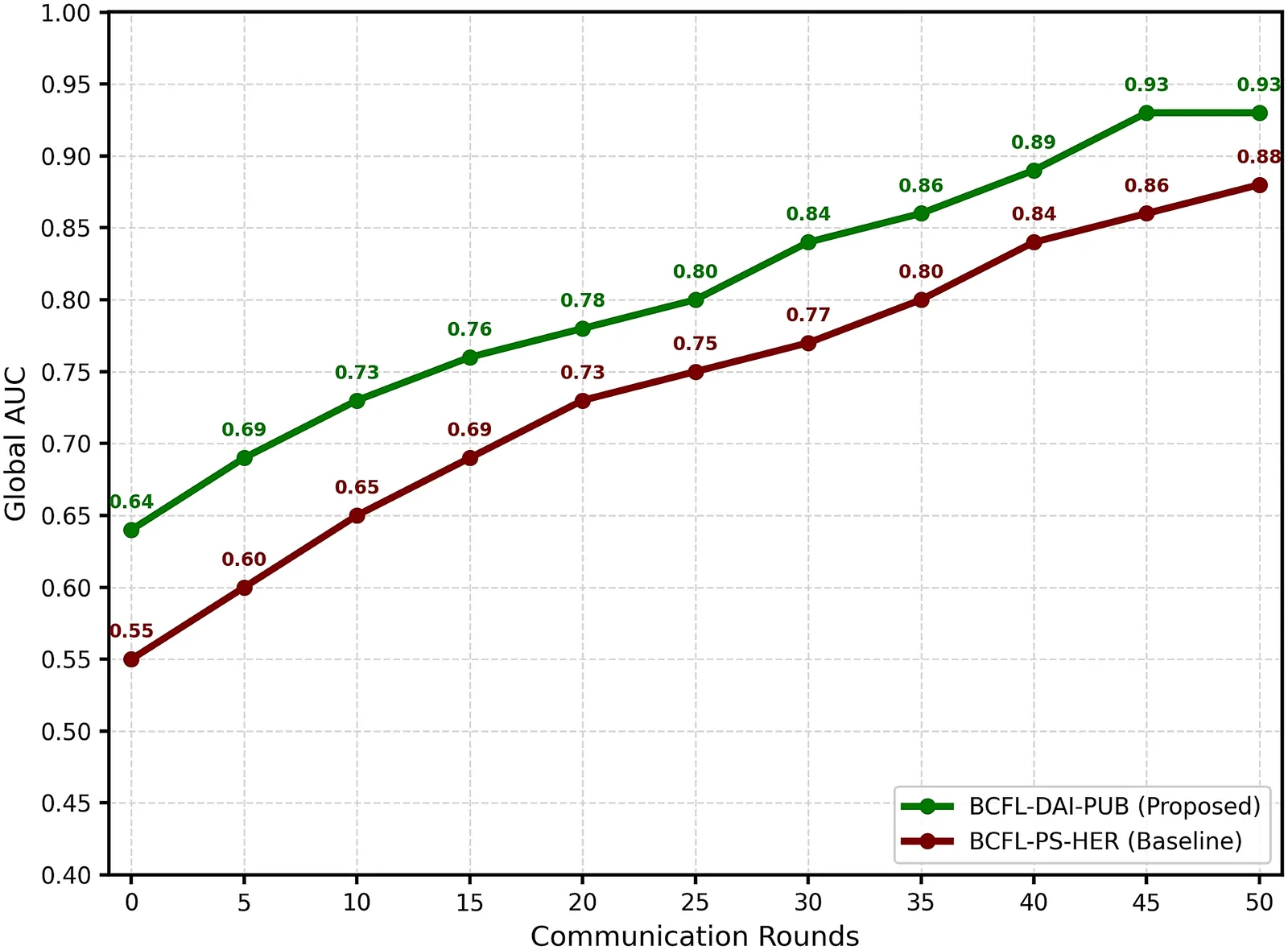
Global model AUC with only honest clients.

#### Reputation-aware robustness against malicious attacks.

When honest, malicious, and free-riding clients participate together, BCFL-DAI-PUB maintains higher global AUC than BCFL-PS-HER throughout training (Fig 10). The proposed framework improves from 0.70 in the initial round to 0.91 by the end of training, despite a brief dip around round 20. In contrast, the baseline rises more slowly, from 0.60 to 0.86. The DAI mechanism improves model stability by isolating the malicious client and suppressing the free-riding participant at an early stage. This control reduces the effect of adversarial or low-quality updates and helps preserve the integrity of the global model.

**Fig 10:**
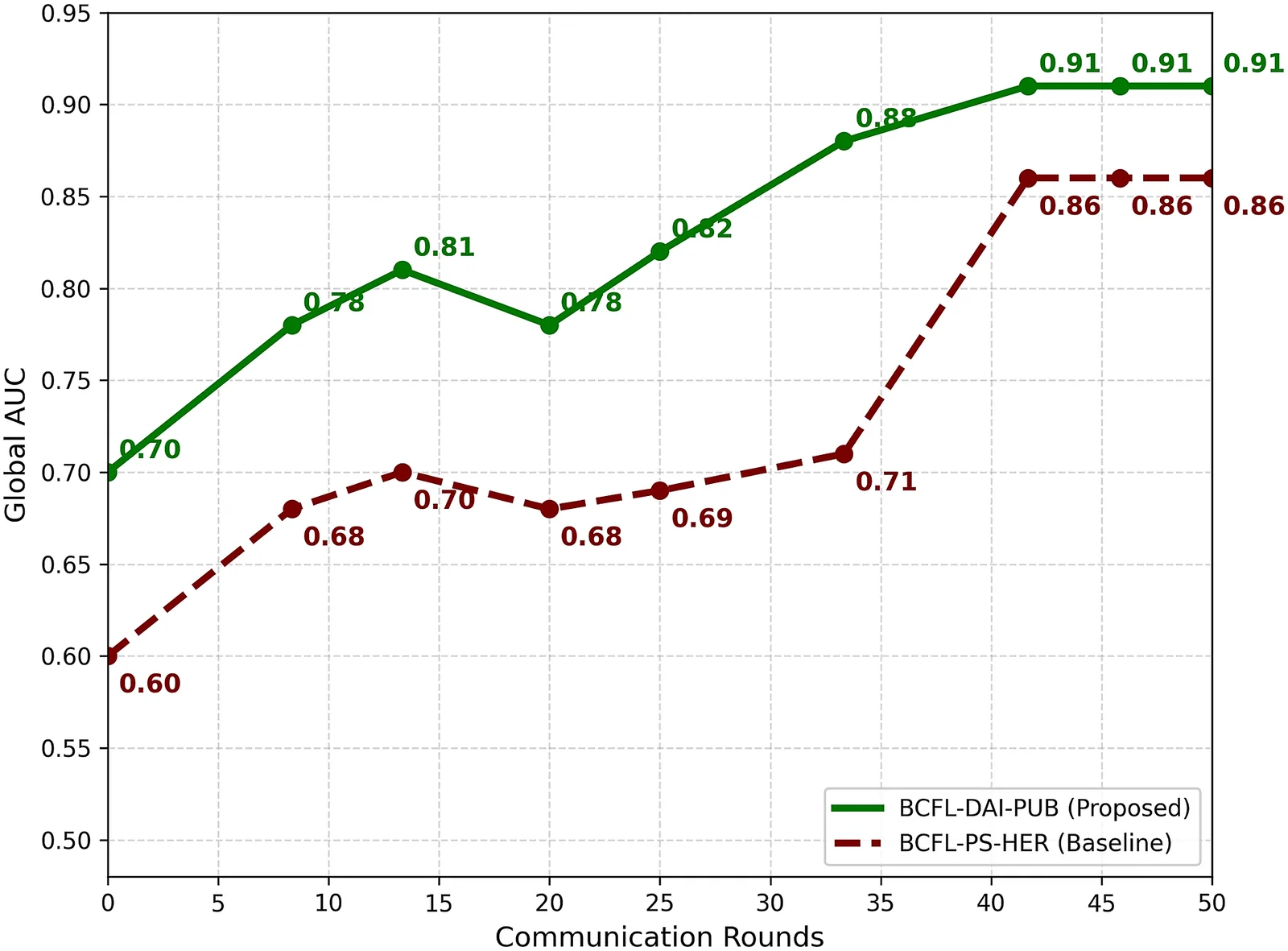
Global model AUC with mixed clients (honest, malicious, and free-rider).

As shown in Fig 10, the performance difference becomes clearly observable immediately after the attack at round 14, thereby highlighting the considerable benefit of a dynamic, contribution-aware defense mechanism over static or incentive-free approaches.

### Assessment of malicious attack resilience and recovery efficiency

This subsection presents evidence that demonstrates the robustness of the proposed **BCFL-DAI-PUB** framework against malicious attacks and allows for evaluating the effectiveness of its recovery process in terms of both model performance and recovery time.

#### Reputation-based mitigation of malicious clients: Performance evaluation.

We evaluate the robustness of BCFL-DAI-PUB against a label-flipping attack by comparing it with BCFL-PS-HER, as shown in Fig 11. To ensure a consistent comparison, the same poisoning interval is applied to both frameworks, beginning at round 14 ($t_{s}=14$) and terminating at round 18 ($t_{e}=18$). The recovery round ($t_{r}$) is identified independently for each framework as the first round at which the model regains its corresponding pre-attack AUC level.

**Fig 11:**
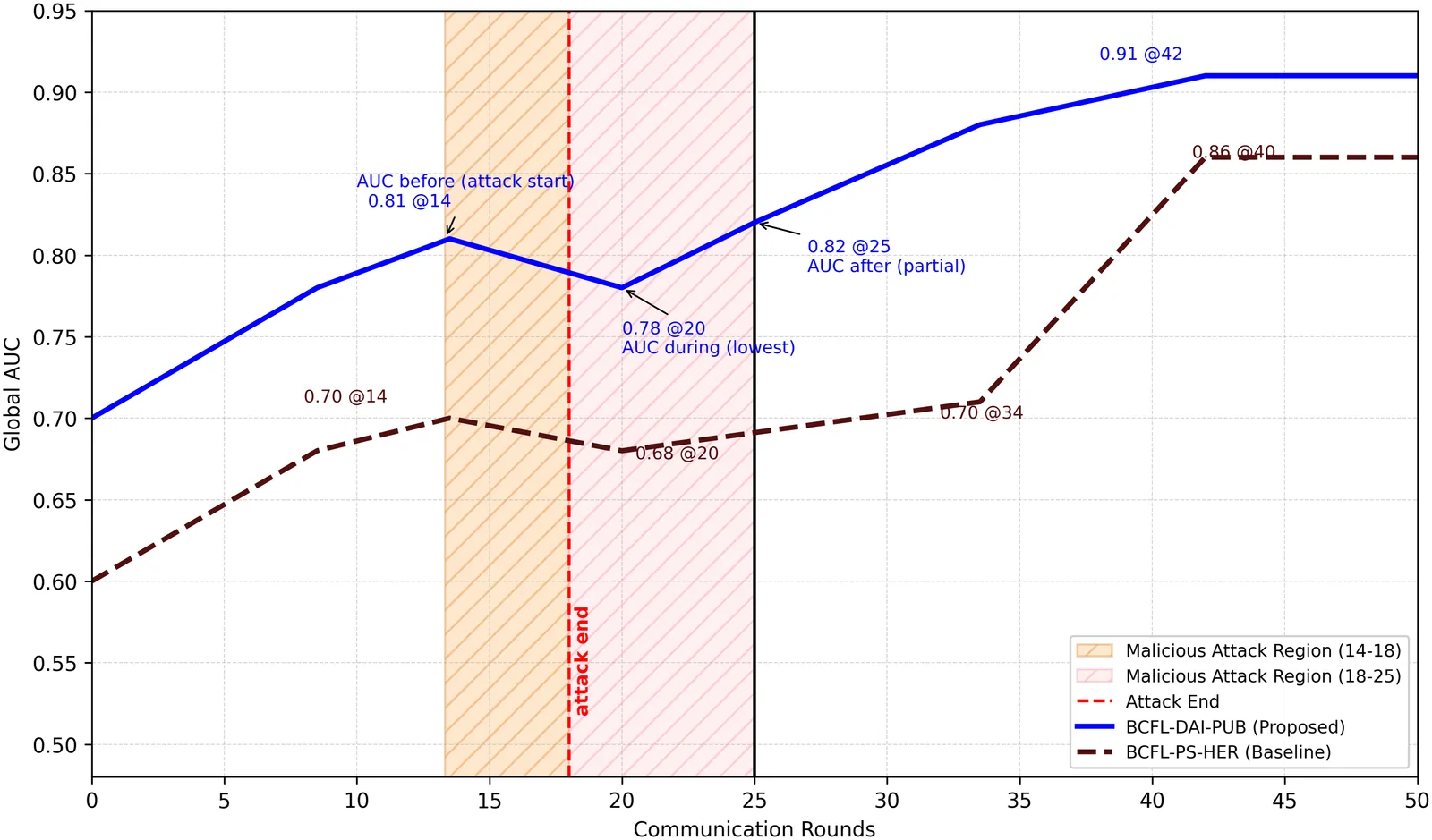
Model robustness under malicious attack.

Two complementary recovery intervals are considered. The attack-onset recovery duration is measured as $t_{r}-t_{s}$ and represents the complete interval from the introduction of malicious behaviour until restoration of pre-attack performance. The post-attack recovery delay is measured as $t_{r}-t_{e}$ and captures only the additional recovery required after the poisoning process has terminated.

For BCFL-PS-HER, the pre-attack AUC is approximately 0.70 at the onset of the attack. Its performance subsequently decreases and remains below the pre-attack level after the attack terminates at round 18. The baseline reaches an AUC of 0.70 again at round 34, which is identified as its recovery round. Therefore, BCFL-PS-HER requires 20 rounds from attack onset to recover (34−14=20), including 16 additional rounds after attack termination (34−18=16). It subsequently improves to an AUC of 0.86 by round 40 and remains stable thereafter.

In contrast, BCFL-DAI-PUB has a pre-attack AUC of approximately 0.81 at the attack onset. Although its AUC temporarily decreases to approximately 0.78 around round 20, the proposed framework restores its pre-attack performance by round 25, reaching an AUC of 0.82. Thus, BCFL-DAI-PUB requires 11 rounds from attack onset to recover (25−14=11) and 7 additional rounds after attack termination (25−18=7). The model subsequently continues to improve, reaching an AUC of 0.91 by round 42 and maintaining this performance through the final communication rounds.

Accordingly, the attack-onset recovery duration is 11 rounds for BCFL-DAI-PUB and 20 rounds for BCFL-PS-HER, whereas the corresponding post-attack recovery delays are 7 and 16 rounds, respectively. The baseline therefore requires approximately 20/11≈1.82× the attack-onset recovery duration of the proposed framework. Since the attack-onset measure captures the complete interval from the first malicious disturbance until restoration of pre-attack performance, it is adopted as the recovery duration used for comparative evaluation in the remainder of this study. The post-attack recovery delay is reported as a complementary measure to indicate the residual recovery required after attack termination. These results indicate that the DAI mechanism limits the effect of malicious updates and enables the global model to restore its pre-attack predictive performance more rapidly than the static-reputation baseline.

#### Recovery speed from poisoning attacks.

Fig 12 clearly demonstrates the proposed BCFL-DAI-PUB’s superior resilience. When a malicious client launches a label-flipping poisoning attack at round 14, BCFL-DAI-PUB fully recovers pre-attack AUC in just 11 additional rounds. In contrast, the static-reputation baseline BCFL-PS-HER requires 20 rounds to recover, making it 1.8× slower.

**Fig 12:**
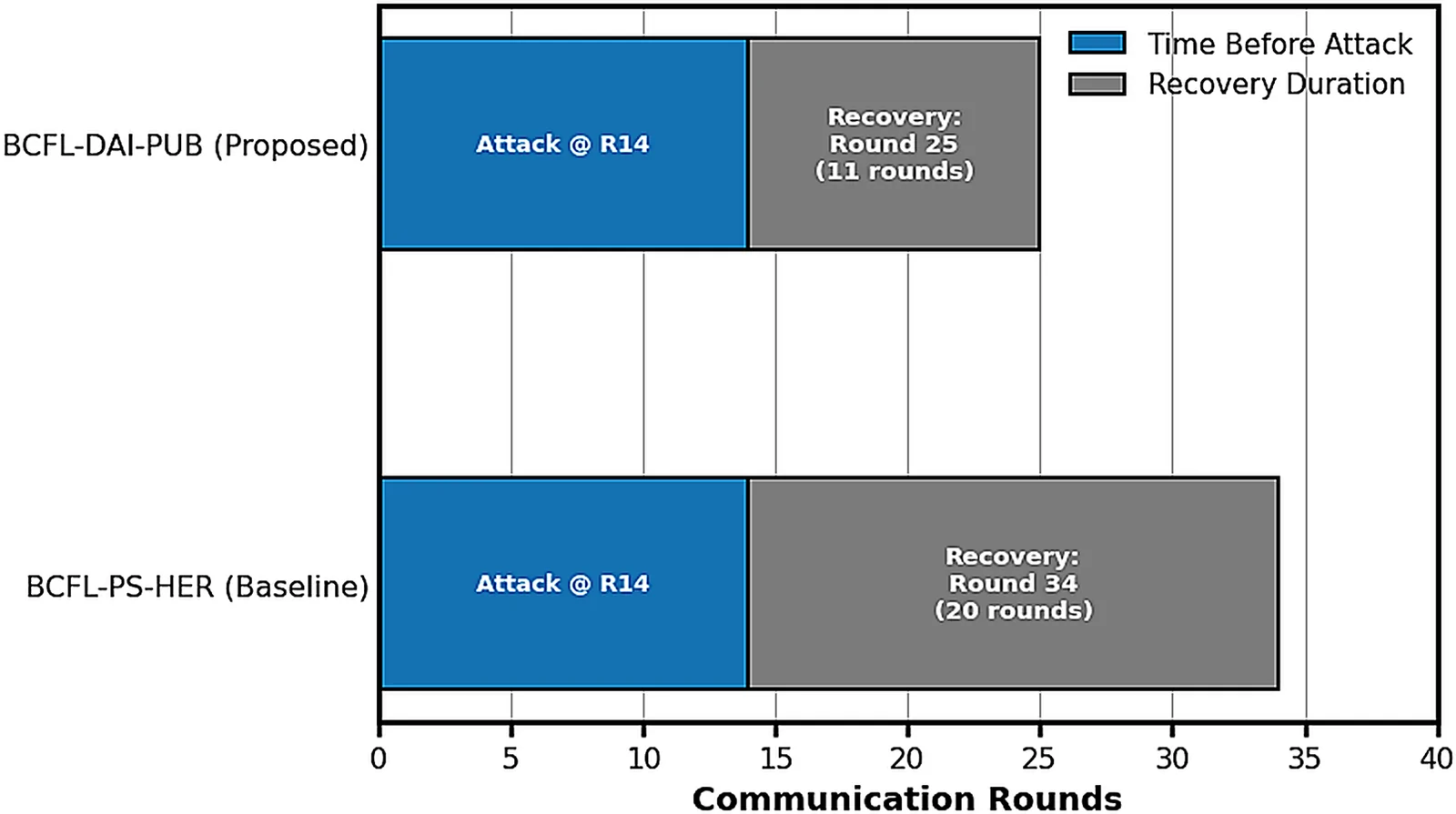
Recovery timeline after poisoning attack at round 14.

### Robustness against gradient inversion attacks (GIA)

To rigorously evaluate the privacy robustness of BCFL-DAI-PUB, we mount the strongest possible Gradient Inversion Attack (GIA) at the final communication round (Round 50, batch size = 1) against all five clients. The attack pipeline integrates the state-of-the-art iGIA and DiffGIA methods [46], supplemented with analytical gradient matching, total-variation and range regularization, randomized diffusion timesteps, and early stopping upon loss convergence. Each attack is executed five times per client, and results are reported as mean ± standard deviation. The following subsection benchmark our framework’s defensive capabilities against the baseline and its quantitative results and analysis

#### Defense settings of proposed against baseline.

The defense capabilities of the proposed and baseline are listed as

- BCFL-PS-HER (Baseline): Homomorphic Encryption protects the transport layer but does not distort the gradient itself. Because GIA requires only access to the mathematical content of the gradient—not the communication channel—HE provides negligible resistance. As a result, the attacker can still accurately optimize the reconstruction objective and recover private samples with high fidelity.
- BCFL-DAI-PUB (Proposed): On the other hand, the proposed approach uses differential privacy at the optimizer level, adding calibrated Gaussian noise directly to the shared gradient. This noise corrupts fine-grained gradient geometry that GIA needs, making the inversion objective unstable and ruining the optimization landscape that is needed to find useful features. Thus, DP systematically disrupts the gradient-data correspondence, rendering high-resolution reconstruction infeasible.

#### Quantitative results and analysis.

Reconstruction quality is assessed using four established tabular metrics (Table 10). Quantitative results are summarized in Table 11 and visualized in Figs 13–15.

**Table 10:** Metrics used to quantify gradient inversion attack success.

Metric	Description
MAE (all features)	Mean absolute error across all 122 features
MAE (Income)	MAE on AMT_INCOME_TOTAL (highest-variance attribute)
Gender Inference Accuracy	Classification accuracy on protected attribute CODE_GENDER
Downstream TARGET AUC	XGBoost AUC trained exclusively on reconstructed records

**Table 11:** Gradient inversion attack results on the home credit default risk dataset (mean ± std over all 5 clients, round 50, batch size = 1).

Method	MAE (all features) ↓	MAE (Income) ↓	Downstream TARGET AUC ↑	Gender Inference ↑
BCFL-PS-HER(Baseline)	4,821 ± 387	12,104 ± 1,208	0.823 ± 0.009	0.93 ± 0.04
BCFL-DAI-PUB (Proposed)	42,105 ± 2,310	91,332 ± 9,104	0.569 ± 0.014	0.52 ± 0.06
Improvement	8.7× worse	7.6× worse	−0.254	−41

**Fig 13:**
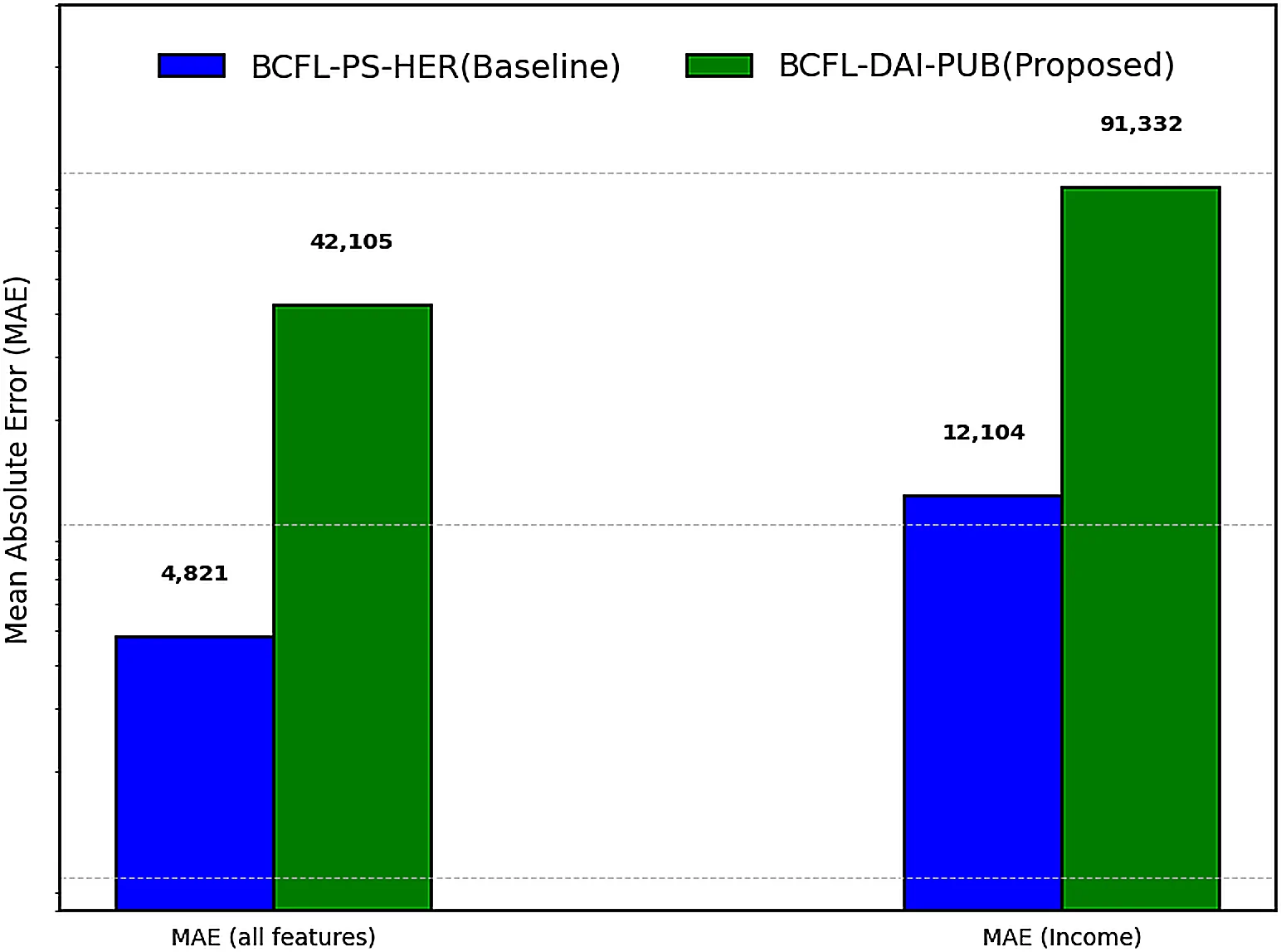
Reconstruction error (MAE) across all features and on income attribute.

**Fig 14:**
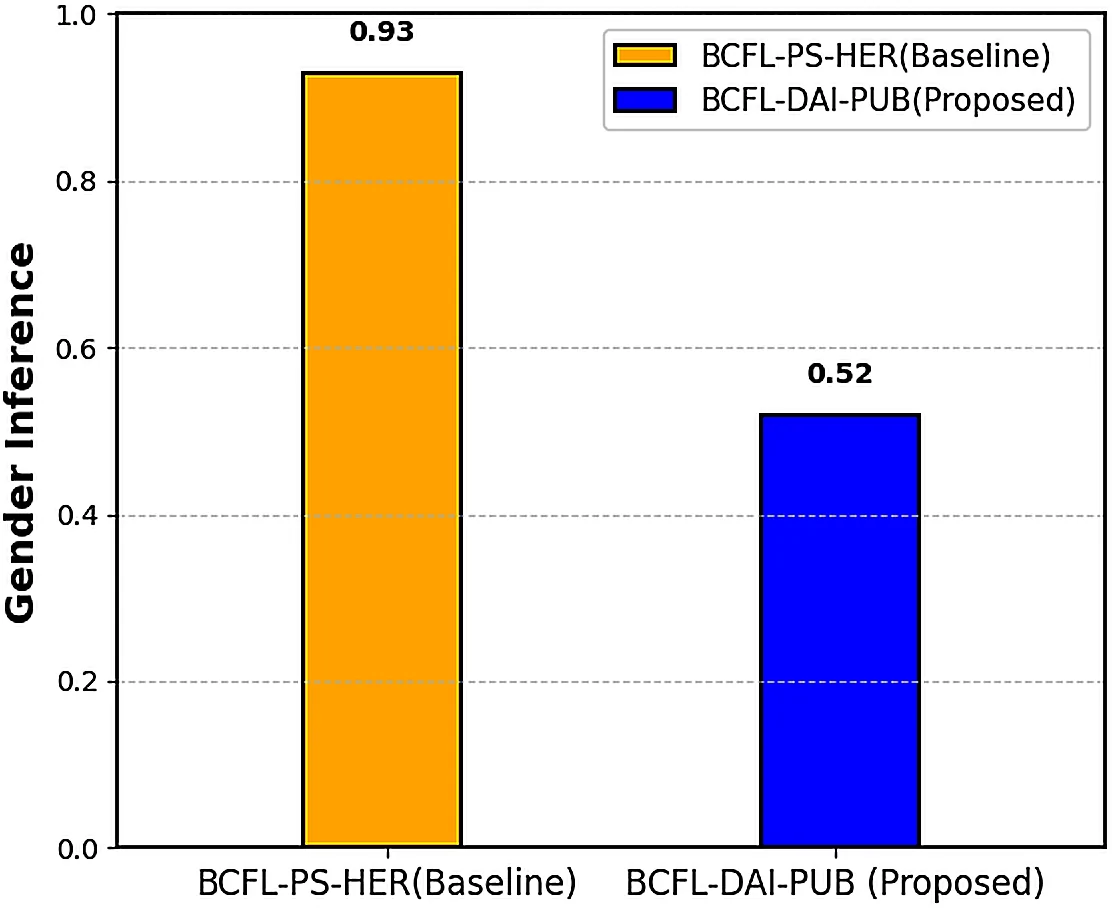
Gender inference accuracy from reconstructed records.

**Fig 15:**
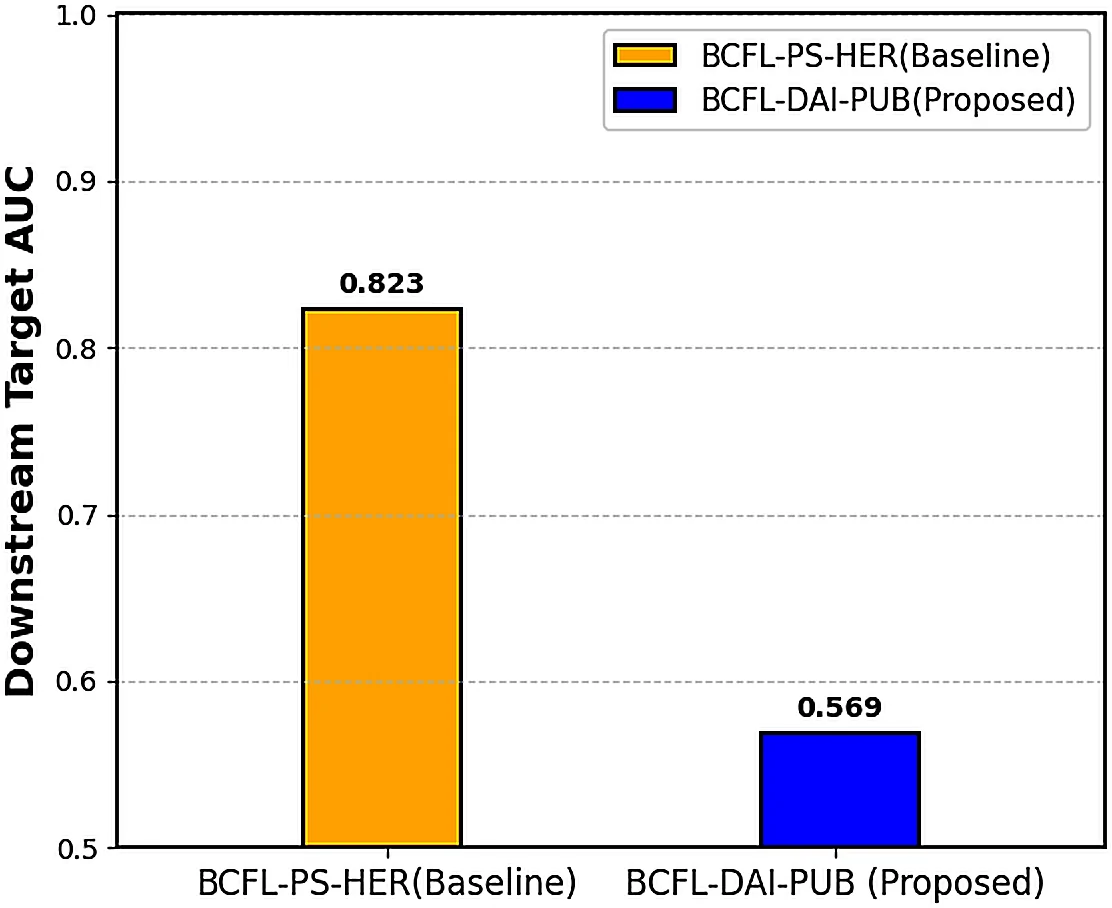
Downstream predictive utility of reconstructed data.

The baseline BCFL-PS-HER suffers severe privacy leakage: annual income is reconstructed with an error of 12*k*, gender is inferred with 93% accuracy, and the reconstructed samples retain strong predictive utility (AUC = 0.823). These results confirm that HE alone does not protect against GIA, as it leaves the gradient signal fully intact.

In contrast, BCFL-DAI-PUB dramatically degrades the attacker’s reconstruction capability. DP noise increases the MAE across all features by 8.7× and increases Income MAE by 7.6×, indicating substantial corruption of the sensitive gradient information. More importantly, the attack loses semantic value: gender inference accuracy collapses by 41 percentage points (down to 0.52), and downstream predictive utility drops by 0.254 AUC. This indicates that the attacker’s reconstructed records are no longer informative or reliable. Overall, these results clearly demonstrate that Differential Privacy is the decisive component enabling strong resistance against Gradient Inversion Attacks, whereas HE-only defenses (as in BCFL-PS-HER) provide little protection. BCFL-DAI-PUB, therefore, ensures significantly improved privacy preservation by fundamentally breaking the gradient-data linkage required for successful inversion.

### Protection against membership inference attacks (MIA)

The baseline BCFL-PS-HER scheme primarily employs Homomorphic Encryption (HE) to keep information private while it is being sent. While HE prevents data exposure in transit, it does not prevent model memorization. Consequently, it offers weak protection against MIAs. Evidence from Fig 16 shows the model remains vulnerable, starting with a high Adv_MIA_ of 0.50 and concluding at 0.22 after 50 rounds, confirming that HE fails to address model-level privacy. Whereas the proposed BCFL-DAI-PUB framework reduces privacy leakage by employing adaptive perturbation during training. The proposed method effectively mitigates overfitting and substantially diminishes the MIA advantage. Evidence from Fig 16 indicates that the model initially exhibits a lower value (0.40 Adv_MIA_) and promptly reduces leakage, reaching an almost negligible Adv_MIA_ of 0.05 by round 50. BCFL-PS-HER focuses on communication security through homomorphic encryption, whereas BCFL-DAI-PUB targets data privacy leakage by dynamically adapting the incentives during training. As a result, BCFL-DAI-PUB shows stronger resistance to inference attacks and malicious participation.

**Fig 16:**
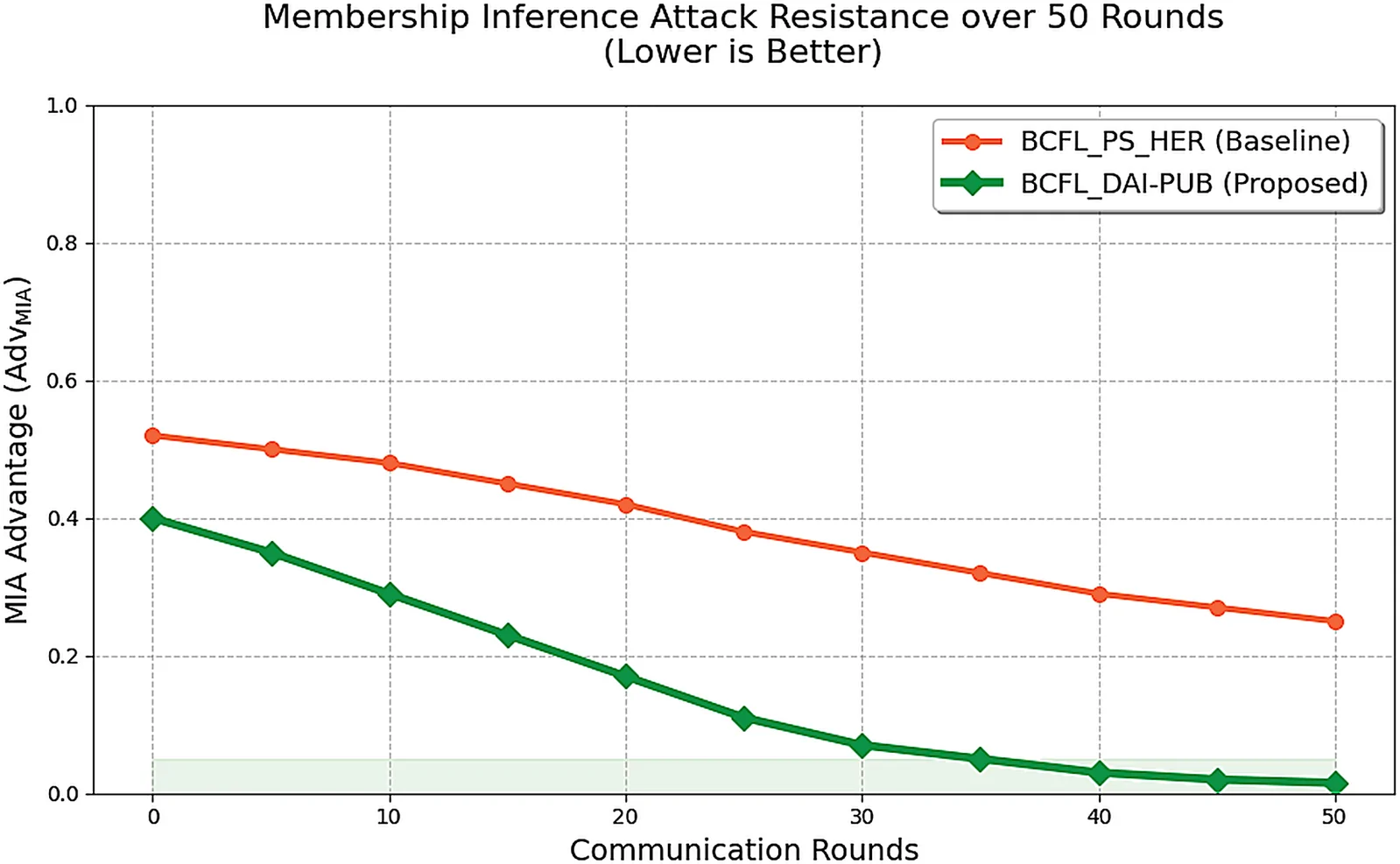
Evolution of membership inference attack (MIA) Advantage across 50 communication rounds.

Using the derived resistance score $R_{MIA}=1-{Adv}_{MIA}$, the corresponding final MIA resistance values are 78% for BCFL-PS-HER and 95% for BCFL-DAI-PUB. Therefore, the proposed BCFL-DAI-PUB framework achieves approximately 1.22× higher MIA resistance than BCFL-PS-HER.

### System overhead and throughput analysis

The proposed framework combines blockchain, federated learning, and an incentive-driven mechanism. We therefore evaluated its overall efficiency in terms of communication overhead, storage overhead, and throughput. Since both BCFL-DAI-PUB and BCFL-PS-HER are blockchain-based frameworks, the reported communication and storage measurements are interpreted as overall system-level overhead comparisons between the complete frameworks, rather than as an isolated assessment of blockchain-specific overhead. The quantitative comparison is summarized in Table 12, while the corresponding trends are illustrated in Figs 17–19.

**Table 12:** Comparison of communication and Storage overhead between the baseline and proposed methods.

Parameter Setting	Communication Overhead (s)					Storage Overhead (MB)			
	Avg.	Avg.	Avg.	Avg.	Avg.	Avg.	Avg.	Avg.	Avg.
	TT	CT	AT	ET	CMT	CNS	CS	MUS	CMS
BCFL-PS-HER (Baseline)	14.01	22.04	4.12	12.00	**52.17**	5.21	2.12	39.27	**46.60**
BCFL-DAI-PUB (Proposed)	13.20	21.58	2.05	11.01	**47.84**	5.01	1.78	34.01	**40.8**

*Abbreviations:* TT:Training time; CT: Consensus Time; AT:Aggregation Time; ET:Evaluation time; CMT: Cumulative Communication Time; CNS: Consensus Size; CS: Chain Size; MUS: Model Update Size; CMS: Cumulative Memory size.

**Fig 17:**
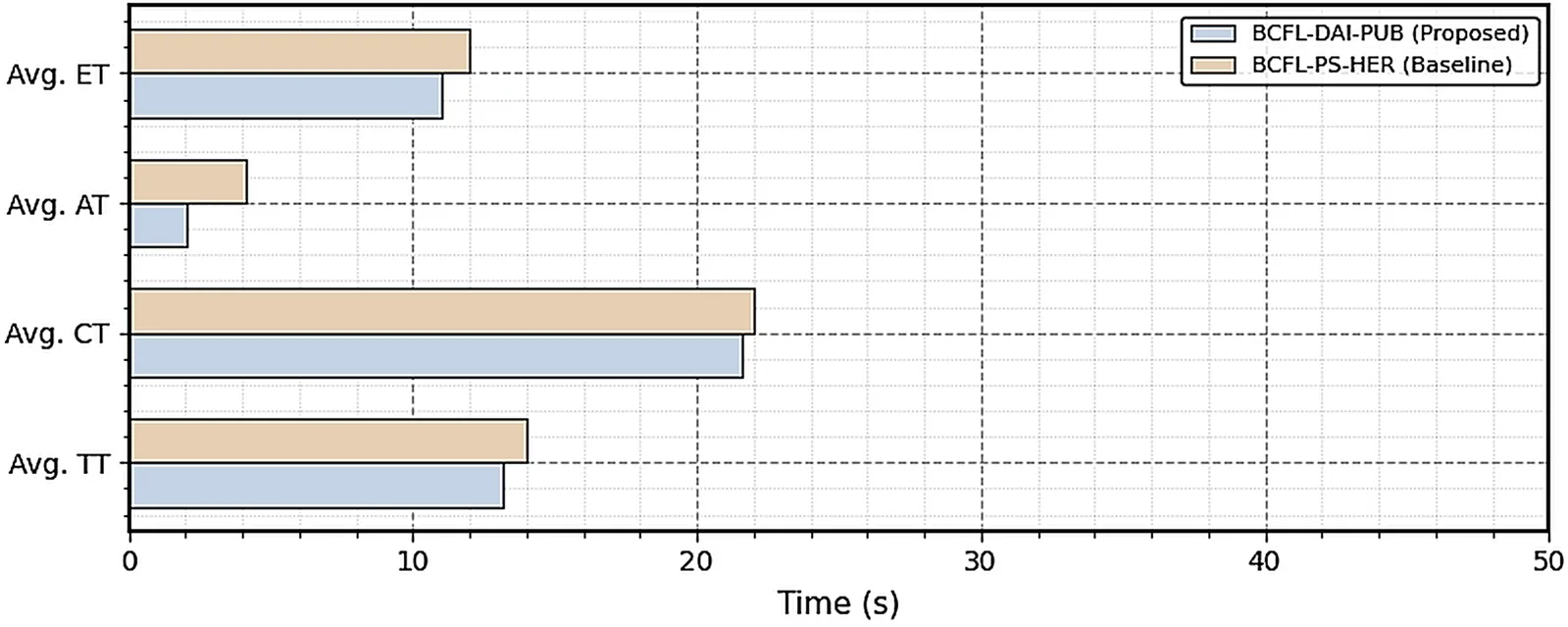
Average communication overhead comparison between BCFL-DAI-PUB and BCFL-PS-HER.

**Fig 18:**
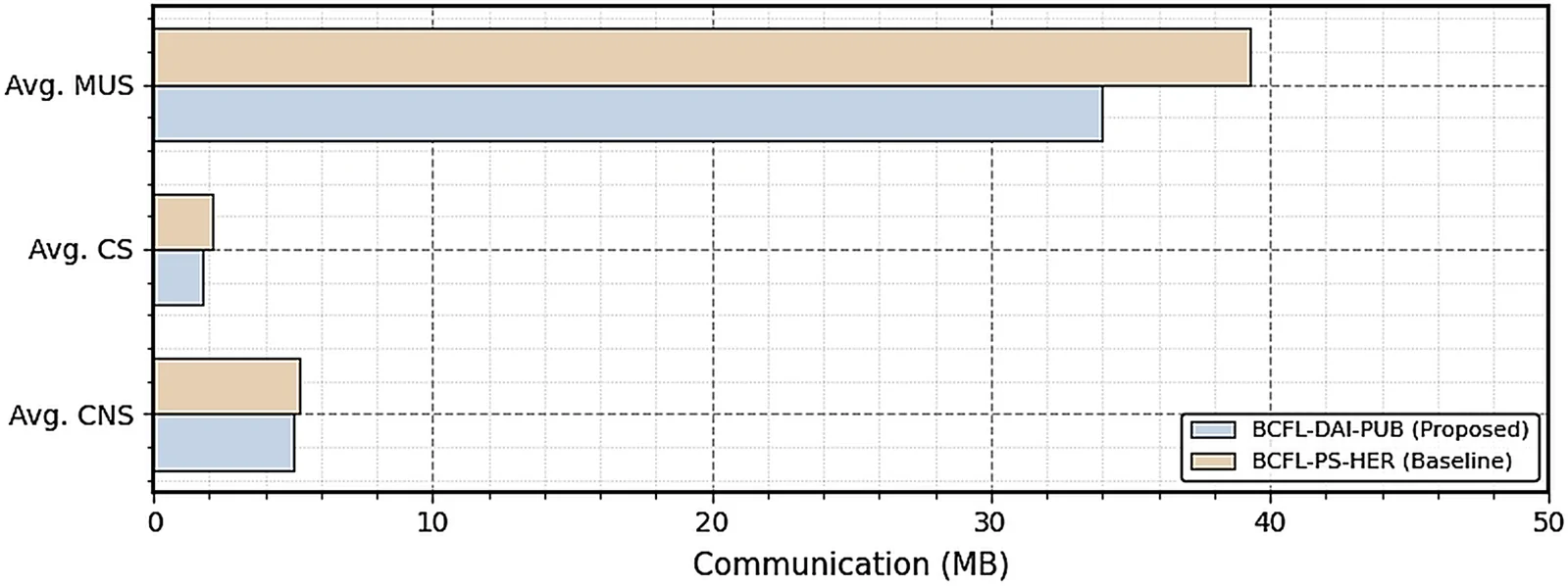
Average storage overhead comparison between BCFL-DAI-PUB and BCFL-PS-HER.

**Fig 19:**
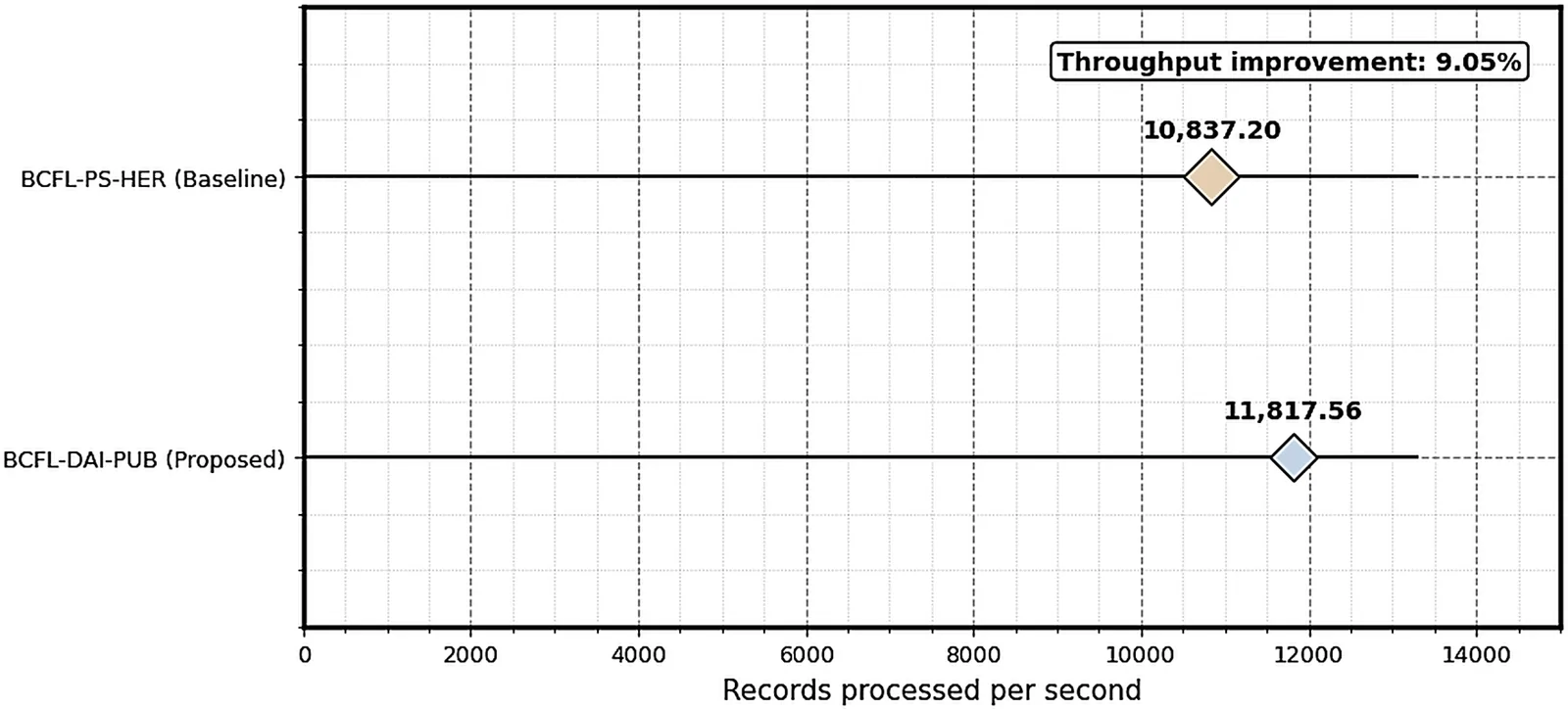
Throughput comparison between BCFL-DAI-PUB and BCFL-PS-HER under the five-client configuration (records processed per second).

#### Communication overhead.

Fig 17 shows that BCFL-DAI-PUB lowers communication overhead by 8.29% relative to BCFL-PS-HER. The cumulative communication overhead decreases from 52.17 s for BCFL-PS-HER to 47.84 s for BCFL-DAI-PUB, indicating that the baseline incurs approximately 1.09× the communication overhead of the proposed framework (52.17/47.84≈1.09). This reduction arises because BCFL-DAI-PUB replaces communication-intensive homomorphic-encrypted exchange with optimizer-level differential privacy, uses SCAFFOLD-based correction to produce more stable local updates, and employs dynamic incentive control to streamline coordination during consensus, aggregation, and evaluation. Together, these design choices reduce the communication burden across the complete training cycle and improve overall communication efficiency.

#### Storage overhead.

Fig 18 shows that BCFL-DAI-PUB reduces memory-related overhead by 12.45% relative to BCFL-PS-HER. The cumulative storage overhead decreases from 46.60 MB for BCFL-PS-HER to 40.8 MB for BCFL-DAI-PUB, indicating that the baseline incurs approximately 1.14× the storage overhead of the proposed framework (46.60/40.8≈1.14). This reduction results from the proposed framework’s hybrid on-chain/off-chain design, which keeps decentralized coordination, smart-contract execution, incentive control, and auditability on-chain while moving bulky learning artifacts off-chain. As a result, the framework reduces on-chain payload size, avoids repeated storage and propagation of large transactions, and lowers blockchain-related storage and processing burden. In addition, BCFL-DAI-PUB replaces ciphertext-heavy update exchange with optimizer-level differential privacy and combines it with SCAFFOLD-based correction and lighter coordination during incentive management and evaluation. Together, these design choices make the proposed framework more memory-efficient than BCFL-PS-HER.

#### Throughput analysis.

To evaluate system processing efficiency, we measured throughput as the number of records processed per second under the same five-client configuration. Fig 19 shows that BCFL-DAI-PUB achieves a throughput of 11,817.56 records/s compared with 10,837.20 records/s for BCFL-PS-HER, corresponding to an improvement of 9.05%. This higher throughput reflects the lower overall computational and communication burden of the proposed framework, enabling more efficient execution of training, consensus, aggregation, and evaluation within the evaluated configuration.

Overall, Figs 17–19 demonstrate lower system-level communication and storage overhead together with improved throughput for BCFL-DAI-PUB under the evaluated five-client setting. Since both frameworks are blockchain-based and the client population is fixed, these results are interpreted as improvements in overall system efficiency within the evaluated configuration.

### Predictive performance evaluation using 10-Fold cross-validation

Table 13 compares the predictive performance of BCFL-PS-HER and the proposed BCFL-DAI-PUB under 10-fold cross-validation. BCFL-DAI-PUB achieves higher values across all four evaluation metrics, improving AUC by 6.31%, Precision by 2.22%, Recall by 3.86%, and F1 Score by 3.09% relative to the baseline. These results show that the proposed framework delivers more reliable classification performance while maintaining a stronger balance between prediction precision and class recovery.

**Table 13:** Performance comparison of the baseline and proposed methods.

Method	AUC	Precision	Recall	F1 Score
BCFL-PS-HER (Baseline)	0.86071±0.00123	0.81916±0.00131	0.72159±0.0021	0.76727±0.00102
BCFL-DAI-PUB (Proposed)	0.91501±0.00007	0.83732±0.00116	0.74946±0.00018	0.79096±0.00008

## State-of-the-art-comparison

Table 14 shows that BCFL-DAI-PUB achieves higher efficiency through two main design choices. First, it uses differential privacy instead of homomorphic encryption, which lowers the communication and computational burden associated with ciphertext-based update exchange. Second, it uses a dynamic incentive mechanism, whereas BCFL-PS-HER relies on a static reputation mechanism. This dynamic design allows the proposed framework to respond more effectively to changing client behaviour and to maintain more efficient coordination during training.

**Table 14:** State-of-the-art comparison of BCFL-PS-HER and BCFL-DAI-PUB across privacy, overhead, and performance dimensions.

Model	Primary Mech.	Reputation Mech.	Recovery Rounds (Malicious)	MIA Resistance (*R*_MIA_)	Communication Overhead	Storage Overhead	Performance
BCFL-PS-HER [31]	HE	Static	20	78%	52.17	46.6	0.86
BCFL-DAI-PUB	ADP	Dynamic	11	95%	47.84	40.8	0.91

**Abbreviations:** HE, Homomorphic Encryption; ADP, Adaptive Differential Privacy; MIA Resistance: $R_{MIA}=1-{Adv}_{MIA}$;

Relative to BCFL-PS-HER, BCFL-DAI-PUB achieves approximately 1.82× faster recovery from malicious disruption and 1.22× higher MIA resistance. In terms of system overhead, BCFL-PS-HER incurs approximately 1.09× the communication overhead and 1.14× the storage overhead of BCFL-DAI-PUB, while the proposed framework achieves approximately 1.06× the predictive performance of the baseline. Taken together, these results demonstrate that BCFL-DAI-PUB strengthens privacy protection, reduces system overhead, improves predictive performance, and enables faster recovery from malicious behaviour. This combined improvement supports more efficient and resilient blockchain-enabled federated credit-risk modelling compared with BCFL-PS-HER.

## Ablation study

The ablation analyzes the individual and collective effects of Blockchain (BC), Federated Learning (FL), the Incentive Mechanism (IM), and Differential Privacy (DP) within the BCFL-DAI-PUB mechanism. As shown in Table 15, robustness in model performance and privacy–utility balance is achieved only when all components are present. Removing any component reduces robustness, increases exposure to reconstruction attacks, and lowers model performance.

**Table 15:** Impact of removing architectural components in BCFL framework.

Architectural Components	Models	Client Participation	Prevents Free Riders	Prevents Malicious Attack	Malicious Attack Recovery	Prevents Gradient Inversion Attack	Prevents Membership Inference Attack	Prevents Single Point Failure	Model Performance
BCFL-DAI-PUB(Proposed)	BC + FL + IM (With DP)	High	✓	✓	✓	✓	✓	✓	0.91
By Removing DP	BC + FL + IM	High	✓	✓	✓	×	×	✓	0.87
By Removing DP and IM	BC + FL	Poor	×	×	×	×	×	✓	0.80
By Removing DP, IM, and BC	FL	Poor	×	×	×	×	×	×	0.76

BC: Blockchain FL: Federated Learning IM: Incentive Mechanism DP: Differential Privacy.

The Dynamic Adaptive Incentive (DAI) mechanism drives the framework by utilizing a dynamic incentive structure to improve model utility and strict privacy adherence simultaneously. By aligning contribution quality with both performance and privacy requirements, DAI effectively resolves the inherent utility–privacy trade-off.

1. **Impact of Removing Differential Privacy (DP)** Removing Differential Privacy (DP) from the framework (leaving BC + FL + IM) retains high client participation and basic malicious defense (due to the Incentive Mechanism, IM), but it immediately sacrifices crucial privacy guarantees. The model is unable to thwart gradient inversion and membership inference attacks, resulting in a performance decline from high to moderate.
2. **Impact of Removing Incentive Mechanism (IM)** Eliminating the incentive mechanism drastically reduces the model’s performance. In the absence of an incentive mechanism, the framework fails to detect malicious participants and free riders, loses the capability to recover from their adverse impacts, and stops to motivate honest clients to participate actively or consistently in the training process.
3. **Impact of Removing Blockchain (BC)** Taking off Blockchain (BC) and leaving only Federated Learning (FL) compromises security and decentralization aspects. The system loses accountability, traceability, and tamper-proof assurances; client participation is inadequate, and model performance declines to a very low level.

The ablation study confirms that the BCFL-DAI-PUB framework functions as a cohesive co-design where every component plays an essential role.

## Managerial and policy implications

The findings show that financial institutions can improve credit-risk modelling without centralizing sensitive customer data. BCFL-DAI-PUB enables collaborative model training across institutions while preserving privacy, limiting malicious influence, and reducing operational overhead. For managers, this means that cross-institution credit-risk assessment no longer requires direct data pooling, which often creates governance, trust, and compliance barriers. The gains in predictive performance, together with lower communication and computational cost, also indicate that the framework is suitable for operational use in settings where both model quality and system efficiency matter.

The study also has clear implications for data governance and compliance practice. By combining blockchain-based auditability, optimizer-level differential privacy, and dynamic incentive control, the framework supports a more accountable and privacy-aware form of collaboration than conventional centralized federated learning. The lower membership inference risk and reduced gradient inversion leakage show that institutions can strengthen data protection while still benefiting from collaborative analytics. These findings support internal policies on privacy-by-design, secure inter-institution model development, and contribution-aware participation governance.

From a policy perspective, the results support auditable and incentive-compatible frameworks for decentralized financial analytics. Faster recovery from malicious clients and stronger discouragement of free-riding show that collaborative learning requires more than technical privacy protection alone. It also requires explicit governance over participant behaviour, contribution quality, and recovery control. The findings therefore support policy frameworks built on three principles: privacy-preserving update sharing, transparent and auditable coordination, and contribution-sensitive participation control. These principles can help regulators and institutional policy-makers design safer collaborative environments for credit-risk assessment and related financial decision systems.

Overall, the study shows that privacy protection, predictive performance, and operational efficiency can be improved together. By strengthening model performance, privacy robustness, recovery behaviour, and system efficiency within a single framework, BCFL-DAI-PUB offers a practical foundation for real-world collaborative credit-risk modelling in decentralized financial environments.

## Conclusion

Privacy-aware credit risk assessment is vital for financial systems, but centralized federated learning presents single points of failure, reducing robustness and data protection. While blockchain-based federated learning enhances decentralization and security, many current methods are still susceptible to inference attacks, slow recovery from malicious clients, and difficulty maintaining strong global model performance while balancing utility and privacy.

To resolve these challenges, this study proposes the BCFL-DAI-PUB framework, which combines blockchain, federated learning, differential privacy, and a dynamic adaptive incentive mechanism. The framework establishes consistent global data representation and performs global feature selection, followed by diffusion-based learning with optimizer-level differential privacy in a blockchain-based federated environment. The incentive mechanism dynamically adjusts client incentives by applying scaffold correction when privacy contribution is high and DP-boost when utility contribution is high, thereby guiding clients toward a stable utility–privacy balance. This adaptive strategy rewards honest clients, limits the influence of malicious and free-riding participants, and improves global model performance while preserving data privacy.

The experimental evaluation shows that the proposed approach performs consistently better than the BCFL-PS-HER baseline across the evaluated settings. The proposed BCFL-DAI-PUB achieves a final AUC of 0.91, improving upon the HE-based BCFL-PS-HER baseline (0.86) by 1.06×. By replacing homomorphic encryption with adaptive differential privacy and combining it with a dynamic incentive mechanism, the proposed framework improves both efficiency and resilience over the baseline, which relies on static reputation-based coordination. BCFL-DAI-PUB recovers from malicious disruption 1.82× faster, improves resistance to membership inference attacks by 1.22×, the baseline incurs approximately 1.09× higher communication overhead and 1.14× higher storage overhead. In addition, the adaptive differential privacy mechanism strengthens privacy protection by lowering the success rate of gradient inversion attacks by a factor of 7.6. These results establish BCFL-DAI-PUB as a reliable, secure, and incentive-driven framework for privacy-aware credit risk assessment in collaborative financial settings.

In conclusion, BCFL-DAI-PUB addresses the major limitations of existing blockchain-based federated learning approaches by jointly improving predictive performance, privacy protection, system efficiency, and resilience to adversarial behaviour. Through the coordinated use of SCAFFOLD-based correction, adaptive differential privacy, and monetary incentives, the framework mitigates malicious interference, discourages free-riding, and maintains a stable privacy–utility balance during training. Overall, these results show that BCFL-DAI-PUB provides a more effective and practical solution for privacy-aware credit risk modelling in decentralized financial environments.
